# Connexin-Based Channel Activity Is Not Specifically Altered by Hepatocarcinogenic Chemicals

**DOI:** 10.3390/ijms222111724

**Published:** 2021-10-29

**Authors:** Kaat Leroy, Alanah Pieters, Axelle Cooreman, Raf Van Campenhout, Bruno Cogliati, Mathieu Vinken

**Affiliations:** 1 Entity of In Vitro Toxicology and Dermato-Cosmetology, Department of Pharmaceutical and Pharmacological Sciences, Vrije Universiteit Brussel, Laarbeeklaan 103, 1090 Brussels, Belgium; kaat.leroy@vub.be (K.L.); alanah.pieters@vub.be (A.P.); axelle.cooreman@vub.be (A.C.); raf.van.campenhout@vub.be (R.V.C.); 2 Department of Pathology, School of Veterinary Medicine and Animal Science, University of São Paulo, Av. Prof. Dr. Orlando Marques de Paiva 87, Cidade Universitária, São Paulo 05508-270, Brazil; bcogliati@usp.br

**Keywords:** connexin, hemichannel, gap junction, genotoxic, non-genotoxic, carcinogen

## Abstract

Connexin-based channels play key roles in cellular communication and can be affected by deleterious chemicals. In this study, the effects of various genotoxic carcinogenic compounds, non-genotoxic carcinogenic compounds and non-carcinogenic compounds on the expression and functionality of connexin-based channels, both gap junctions and connexin hemichannels, were investigated in human hepatoma HepaRG cell cultures. Expression of connexin26, connexin32, and connexin43 was evaluated by means of real-time reverse transcription quantitative polymerase chain reaction analysis, immunoblot analysis and in situ immunostaining. Gap junction functionality was assessed via a scrape loading/dye transfer assay. Opening of connexin hemichannels was monitored by measuring extracellular release of adenosine triphosphate. It was found that both genotoxic and non-genotoxic carcinogenic compounds negatively affect connexin32 expression. However, no specific effects related to chemical type were observed at gap junction or connexin hemichannel functionality level.

## 1. Introduction

Gap junctions allow for direct intercellular communication between neighboring cells via diffusion of small and hydrophilic molecules and ions. This flux, called gap junction intercellular communication (GJIC), is crucial for the preservation of normal homeostasis [1]. In liver physiology specifically, GJIC plays an essential role during various liver-specific functions, such as ammonia detoxification [2], biotransformation of xenobiotic compounds [3,4,5], albumin secretion [2], glycogenolysis [6,7,8], and secretion of bile [9,10]. Gap junctions are built up by two connexin hemichannels of adjacent cells, which are each themselves constituted by six connexin (Cx) proteins. Thus far, 21 mammalian connexin proteins have been discovered [11]. Because of their cell-specific expression, not all connexin proteins are found in the liver. Indeed, Cx32 and small quantities of Cx26 are expressed by healthy hepatocytes, while Cx43 is typically found in non-parenchymal cells [12,13,14,15,16]. However, during liver disease, including cancer, hepatic Cx43 expression increases at the expense of Cx32 and Cx26 [17,18,19,20,21].

Chemicals are major inducers of cancer and act via genotoxic and/or non-genotoxic mechanisms [22]. Genotoxicity denotes any insult to the genetic information, such as mutagenicity, clastogenicity, and aneugenicity, and can occur in virtually all cell types [23]. By contrast, non-genotoxic carcinogenicity is a tissue-specific and species-specific process that encompasses a wide diversity of mechanisms [24]. In the 1990s, considerable attention has been paid to the inhibition of GJIC as a potential feature of non-genotoxic carcinogenic (NGTX) chemicals [25,26,27]. Throughout that research, focus has been put on the liver, which is not a surprise, given its key function as a detoxification hub in the organism, hence representing a major target of toxicity [28]. However, these reports typically studied the effects of carcinogenic chemicals either at the expression level or at the functional level. For this reason, and thus in order to capture the full scenario of actions, it is of utmost importance to simultaneously consider the impact of carcinogenic chemicals at all relevant levels. This actually defines the rationale and scope of the present study. Human hepatoma HepaRG cells are exposed to a set of well-known genotoxic carcinogenic (GTX) chemicals, NGTX chemicals, and non-carcinogenic (NC) chemicals (Table 1). Their effects are investigated at the transcriptional (real-time reverse transcription quantitative polymerase chain reaction analysis (RT-qPCR)), translational (immunoblot and in situ immunostaining analyses) and functional (scrape loading/dye transfer analysis and measurement of extracellular adenosine triphosphate (ATP) release) levels.

## 2. Results

### 2.1. Connexin Protein Expression in HepaRG Cells Compared to Primary Human Hepatocytes

**Figure 1:**
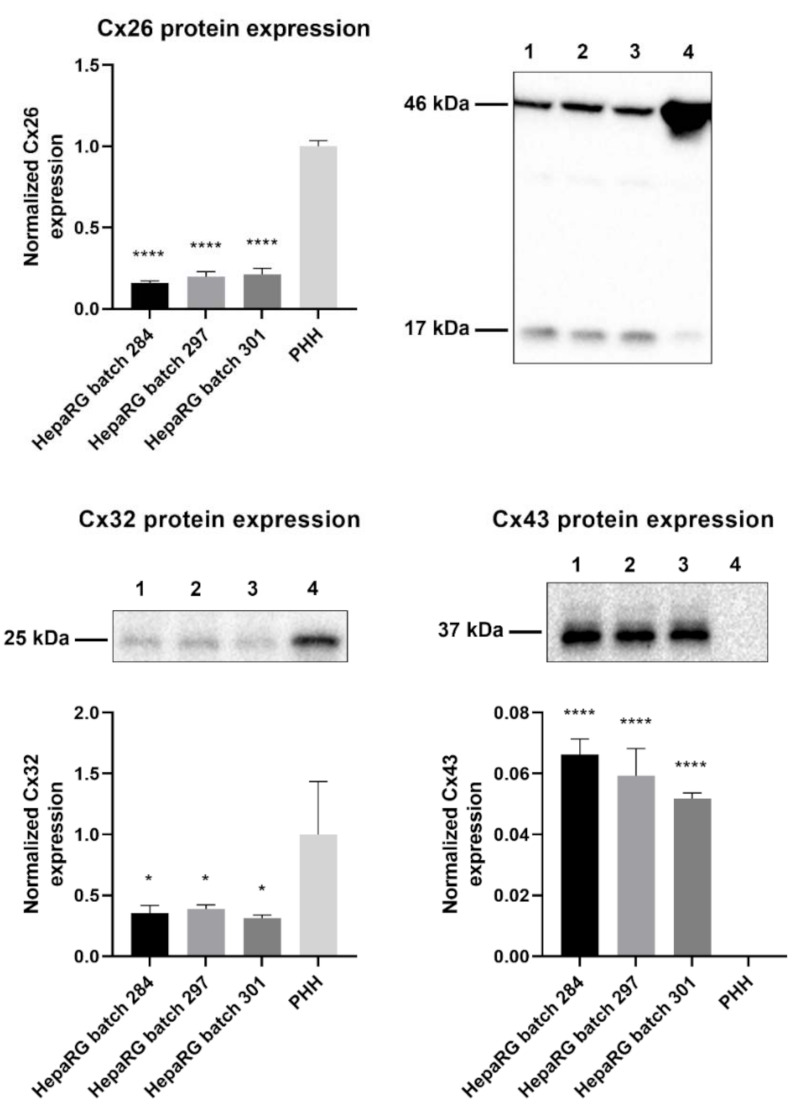
Cx26, Cx32, and Cx43 protein expression in human hepatoma HepaRG cells compared to primary human hepatocytes (PHH). Proteins were extracted from human hepatoma HepaRG cells (*n* = 3 and N = 3) and PHH (*n* = 1 and N = 3) and immunoblotting was performed. Densiometric quantitative data were obtained via Image Lab 6.0.1 software (Bio-Rad, Hercules, CA, USA). Data were normalized to the total protein loading (Figure S1) and expressed as a ratio to PHH (if the target was expressed in PHH). Significant differences compared to PHH were calculated with a parametric one-way analysis of variance (ANOVA) followed by a Dunnett’s post-hoc test to correct for multiple comparisons. Data are expressed as mean ± standard deviation with * *p* ≤ 0.05 and **** *p* ≤ 0.0001. (1 = HepaRG batch 284; 2 = HepaRG batch 297; 3 = HepaRG batch 301; 4 = PHH).

Primary hepatocytes, especially primary human hepatocytes (PHH), are often considered to be the gold standard for toxicity testing [31]. However, previous in vitro carcinogenicity testing has proven that HepaRG cells are better at distinguishing GTX chemicals from NGTX and NC chemicals via transcriptomics analysis than primary rat hepatocytes [32]. The connexin protein expression pattern of PHH was compared to that of human hepatoma HepaRG cells via immunoblot analysis to characterize the connexin expression of the chosen cell model. The results indicated that Cx26 and Cx32 are still being expressed in HepaRG cells, despite their tumorigenic origin (Figure 1). Cx26 was detected at two different molecular weights during immunoblot analysis, more specifically at 17 kDa and 46 kDa. The band at 46 kDa was presumed to be a dimer [33]. Hence, both bands were incorporated during densiometric analysis. Cx43 was expressed by HepaRG cells but not by PHH (Figure 1).

### 2.2. Effects of (Non)-Carcinogenic Chemicals on Connexin Gene Expression

Cx32 accounts for 90% of the connexins expressed in human hepatocytes, while Cx26 makes up 5% [34]. Non-parenchymal cells, including Kupffer cells and stellate cells, mainly express Cx43 [12,14,16,34,35]. This expression pattern changes during liver disease [36]. Overall, Cx32 and Cx26 expression is believed to be downregulated at the expense of de novo Cx43 expression in human hepatocytes upon pathology [21,36]. Additionally, Cx43 expression is upregulated in non-parenchymal cells in many liver disorders [21,36]. While some hepatocellular carcinoma (HCC) studies support this connexin expression switching pattern, various observations have been reported, in different HCC models [18,21,36,37,38,39].

**Figure 2:**
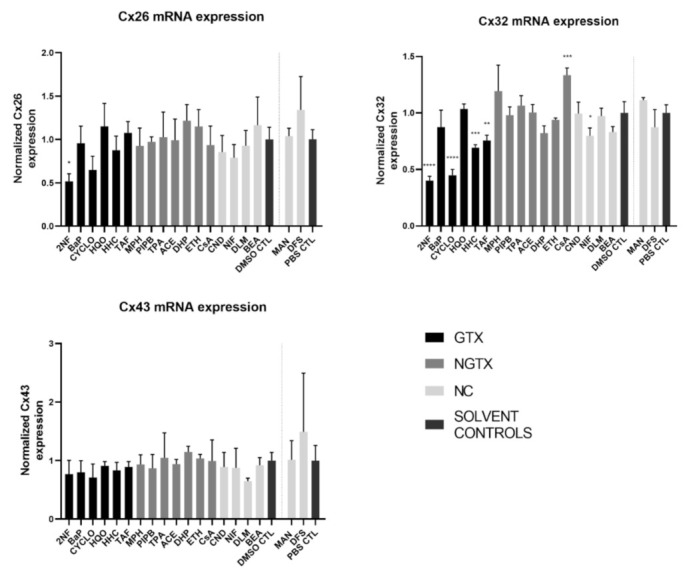
Connexin (Cx) gene expression in human hepatoma HepaRG cells exposed to GTX, NGTX, and NC chemicals. Human hepatoma HepaRG cells (*n* = 3 and N = 2) were exposed to GTX, NGTX, and NC chemicals for 72 h and compared to their respective solvent control (DMSO CTL or PBS CTL). Total RNA was extracted, and expression levels were measured by means of real-time reverse transcription quantitative polymerase chain reaction (RT-qPCR) analysis. Data were analyzed with the 2^−ΔΔCt^ formula in qbase+ (Biogazelle, Belgium). Significance was calculated with a parametric one-way ANOVA followed by a Dunnett’s post-hoc test to correct for multiple comparisons. Data are expressed as mean ± standard deviation with * *p* ≤ 0.05 ** *p* ≤ 0.01 *** *p* ≤ 0.001 and **** *p* ≤ 0.0001.

RT-qPCR analysis was performed in this study to investigate the effects of various chemicals on connexin mRNA expression. HepaRG cells were exposed to GTX, NGTX, and NC chemicals and compared to their respective solvent control, namely dimethyl sulfoxide (DMSO) or phosphate-buffered saline (PBS) (Figure 2).

The GTX chemical 2-nitrofluorene (2NF) was the only chemical that significantly reduced Cx26 mRNA levels. Four GTX chemicals, namely 2NF, cyclophosphamide (CYCLO), hydrazine dihydrochloride (HHC), and 2-acetylaminofluorene (TAF) and the NC chemical nifedipine (NIF), lowered Cx32 expression, while cyclosporine A (CsA), an NGTX chemical, positively affected Cx32. No changes were induced by any of the chemicals on Cx43 mRNA expression levels.

### 2.3. Effects of (Non)-Carcinogenic Chemicals on Connexin Protein Expression and Localization

**Figure 3:**
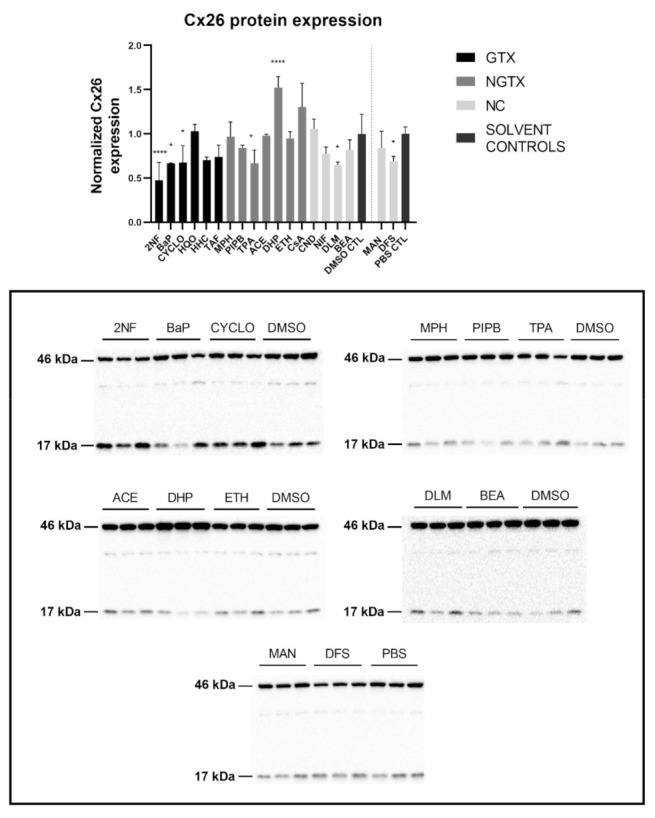
Cx26 protein expression in human hepatoma HepaRG cells exposed to GTX, NGTX, and NC chemicals. Human hepatoma HepaRG cells (*n* = 3 and N = 1) were exposed to (non)-carcinogenic chemicals for 72 h. Densiometric quantitative data were obtained via Image Lab 6.0.1 software (Bio-Rad, Hercules, CA, USA). Data were normalized to the total protein loading (Figure S2) according to Bio-Rad’s instructions [40] and expressed as a ratio to their respective solvent control (DMSO CTL or PBS CTL). Significant difference compared to the solvent control (DMSO CTL or PBS CTL) was calculated with a parametric one-way ANOVA followed by a Dunnett’s post-hoc test to correct for multiple comparisons. Data are expressed as mean ± standard deviation with * *p* ≤ 0.05 and **** *p* ≤ 0.0001. Only blots containing significantly affected expression patterns are shown.

**Figure 4:**
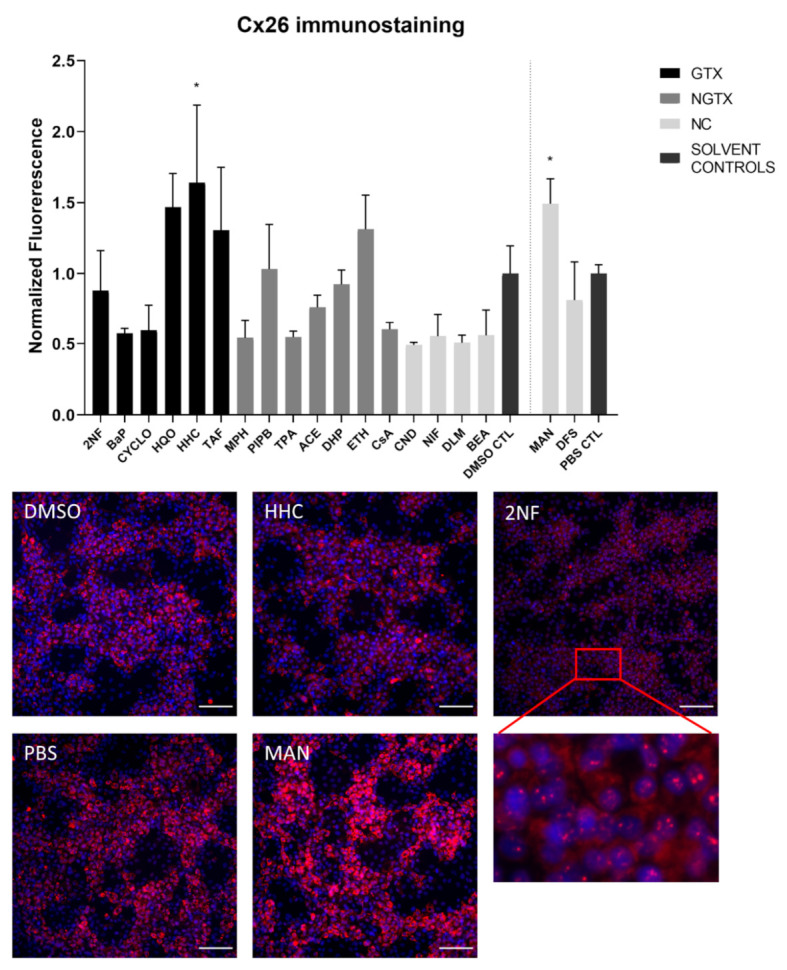
Cx26 protein localization in human hepatoma HepaRG cells exposed to GTX, NGTX, and NC chemicals. Human hepatoma HepaRG cells (*n* = 1 and N = 1; at least three images per well) were exposed to GTX, NGTX, and NC chemicals for 72 h. The area and intensity of the fluorescent signal was quantified via ImageJ software (version 1.52p, Bethesda, MD, USA) and normalized to the fluorescent signal of the respective solvent control (DMSO CTL or PBS CTL). Significant difference compared to the solvent control was calculated with a parametric one-way ANOVA followed by a Dunnett’s post-hoc test to correct for multiple comparisons. Data are expressed as mean ± standard deviation with * *p* ≤ 0.05. Only images of significantly affected chemicals and 2NF are shown. Scale bar = 100 µM, 20× objective.

Protein expression was evaluated by means of immunoblot analysis and in situ immunostaining. Cx26 was again detected at both 17 kDa and 46 kDa during immunoblot analysis. Both bands were incorporated during densiometric analysis. In contrast to its mRNA expression, Cx26 protein levels were significantly downregulated by 3 GTX chemicals, namely 2NF, benzyl alcohol (BEA) and CYCLO, the NGTX chemical tetradecanoyl phorbol acetate (TPA) and the 2 NC chemicals, *D,L*-menthol (DLM) and diclofenac sodium (DFS) (Figure 3). The NGTX chemical diethylhexyl phthalate (DHP) was the only chemical to upregulate Cx26 protein expression. Immunostaining on the other hand showed increased Cx26 signals caused by HHC and mannitol (MAN) compared to their respective solvent control (Figure 4 and Figure S3). All other chemicals, except hydroquinone (HQO), TAF, piperonyl butoxide (PIPB) and ethanol (ETH), tended to downregulate the Cx26 signal although this effect was not significant.

**Figure 5:**
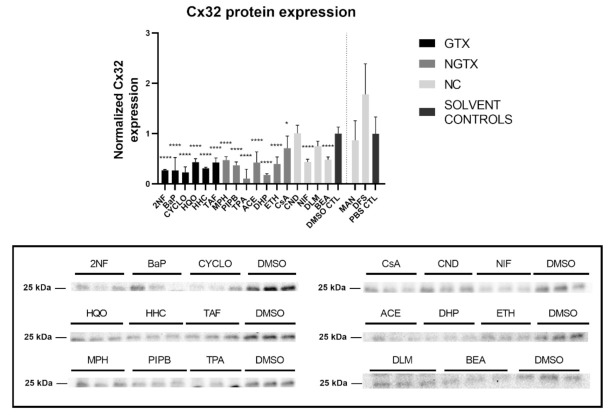
Cx32 protein expression in human hepatoma HepaRG cells exposed GTX, NGTX, and NC chemicals. Human hepatoma HepaRG cells (*n* = 3 and N = 1) were exposed to GTX, NGTX, and NC chemicals for 72 h. Densiometric quantitative data were obtained via Image Lab 6.0.1 software (Bio-Rad, Hercules, CA, USA). Data were normalized to the total protein loading (Figure S4) according to Bio-Rad’s instructions [40] and expressed as a ratio to their respective solvent control (DMSO CTL or PBS CTL). Significant difference compared to the solvent control (DMSO CTL or PBS CTL) was calculated with a parametric one-way ANOVA followed by a Dunnett’s post-hoc test to correct for multiple comparisons. Data are expressed as mean ± standard deviation with * *p* ≤ 0.05 and **** *p* ≤ 0.0001. Only blots containing significantly affected expression patterns are shown.

**Figure 6:**
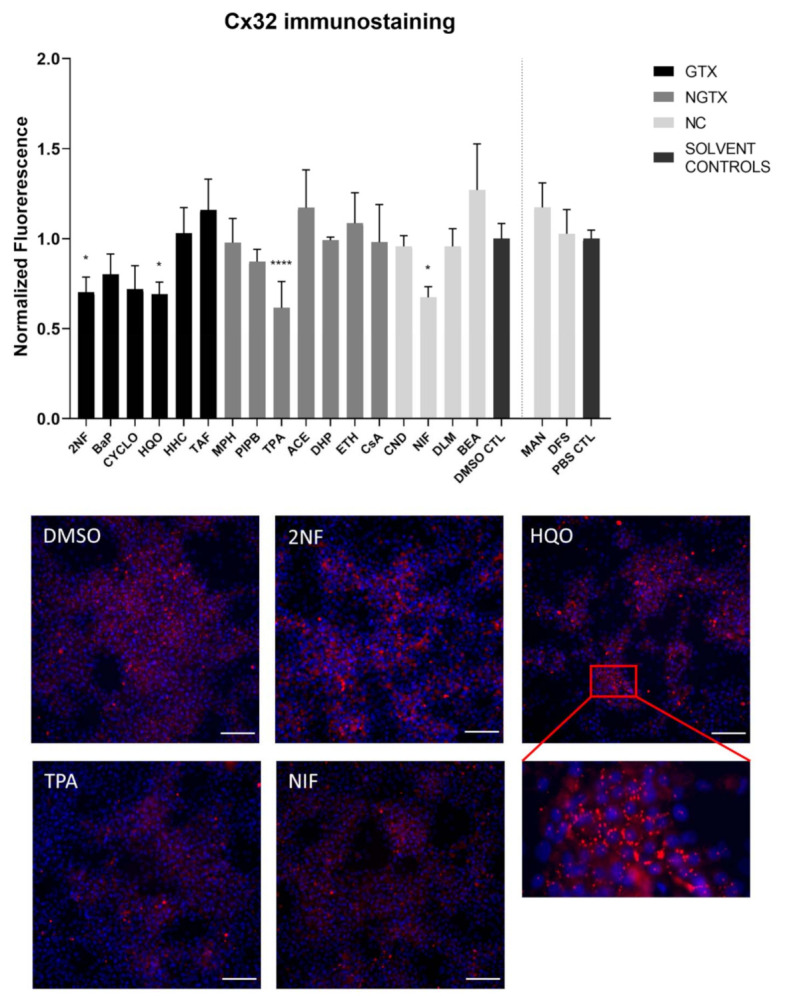
Cx32 protein localization in human hepatoma HepaRG cells exposed GTX, NGTX, and NC chemicals. Human hepatoma HepaRG cells (*n* = 1 and N = 1; at least 3 images per well) were exposed to GTX, NGTX, and NC chemicals for 72 h. The area and intensity of the fluorescent signal was quantified via ImageJ software (version 1.52p, Bethesda, MD, USA) and normalized to the fluorescent signal of the respective solvent control (DMSO CTL or PBS CTL). Significant difference compared to the solvent control was calculated with a parametric one-way ANOVA followed by a Dunnett’s post-hoc test to correct for multiple comparisons. Data are expressed as mean ± standard deviation with * *p* ≤ 0.05 and **** *p* ≤ 0.0001. Only images of significantly affected chemicals are shown. Scale bar = 100 µM, 20× objective.

Furthermore, all GTX and NGTX chemicals evoked a decrease of Cx32 protein expression according to the immunoblot analysis (Figure 5), yet the same was seen for 2 NC chemicals, namely NIF and BEA. Although immunoblot analysis indicated a significant drop in Cx32 expression for all GTX and NGTX chemicals, this was not mirrored by the immunostaining analysis, in which only 2NF, HQO, TPA, and NIF significantly lowered the Cx32 signal (Figure 6 and Figures S5–S8).

**Figure 7:**
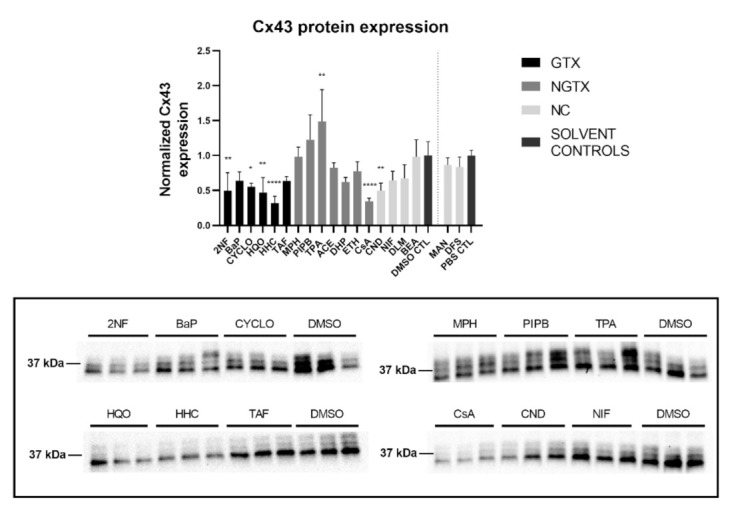
Cx43 protein expression in human hepatoma HepaRG cells exposed to GTX, NGTX and NC chemicals. Human hepatoma HepaRG cells (*n* = 3 and N = 1) were exposed to GTX, NGTX, and NC chemicals for 72 h. Densiometric quantitative data were obtained via Image Lab 6.0.1 software (Bio-Rad, Hercules, CA, USA). Data were normalized to the total protein loading (Figure S9) according to Bio-Rad’s instructions [40] and expressed as a ratio to their respective solvent control (DMSO CTL or PBS CTL). Significant difference compared to the solvent control was calculated with a parametric one-way ANOVA followed by a Dunnett’s post-hoc test to correct for multiple comparisons. Data are expressed as mean ± standard deviation with * *p* ≤ 0.05 ** *p* ≤ 0.01 and **** *p* ≤ 0.0001. Only blots containing significantly affected expression patterns are shown.

**Figure 8:**
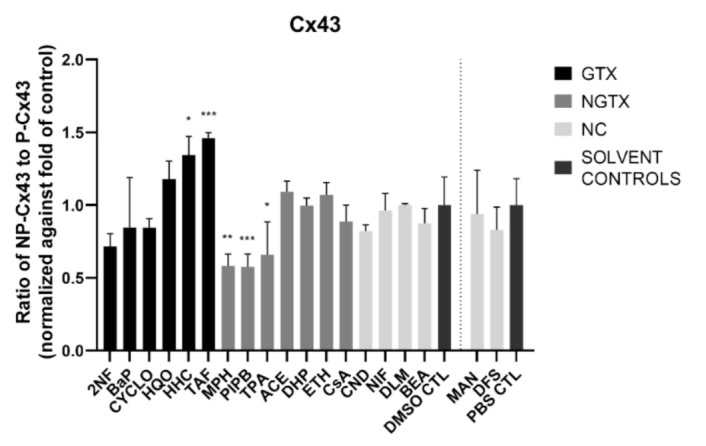
Ratio of the non-phosphorylated (NP-Cx43) isoforms against the two phosphorylated isoforms of Cx43 (P-Cx43). Human hepatoma HepaRG cells (*n* = 3 and N = 1) were exposed to (non)-carcinogenic chemicals for 72 h. Densiometric quantitative data were obtained via Image Lab 6.0.1 software (Bio-Rad, Hercules, CA, USA). Data were normalized to the total protein loading (Figure S9) and a ratio of NP-Cx43 to P-Cx43 was calculated per compound. The ratios were normalized to the fold change of their respective solvent control (DMSO CTL or PBS CTL). Significant difference compared to the solvent control was calculated with a parametric one-way ANOVA followed by a Dunnett’s post-hoc test to correct for multiple comparisons. Data are expressed as mean ± standard deviation with * *p* ≤ 0.05 ** *p* ≤ 0.01 and *** *p* ≤ 0.001.

**Figure 9:**
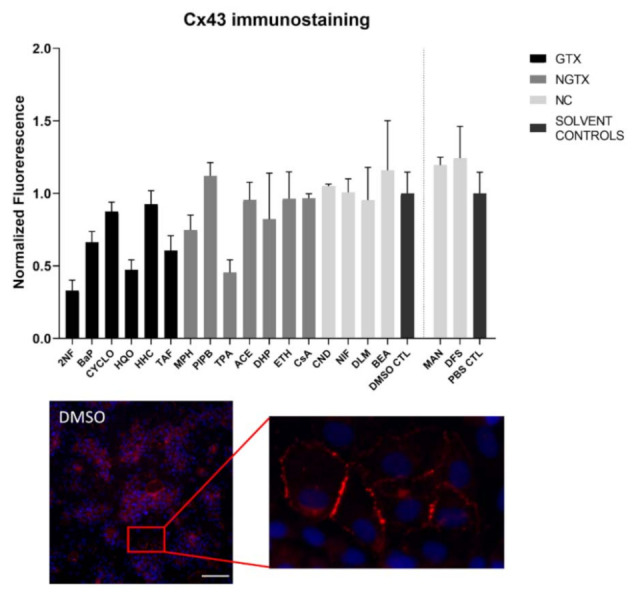
Cx43 protein localization in human hepatoma HepaRG cells exposed to GTX, NGTX and NC chemicals. Human hepatoma HepaRG cells (*n* = 1 and N = 1; at least 3 images per well) were exposed to GTX, NGTX, and NC chemicals for 72 h. The area and intensity of the fluorescent signal was quantified via ImageJ software (version 1.52p, Bethesda, MD, USA) and normalized to the fluorescent signal of the respective solvent control (DMSO CTL or PBS CTL). Significant difference compared to the solvent control was calculated with a non-parametric Kruskal-Wallis test followed by a Dunn’s multiple comparison test. Data are expressed as mean ± standard deviation. No significant changes were measured. Scale bar = 100 µM, 20× objective.

Cx43 immunoblots typically displayed three different bands around 37 kDa due to post-translational phosphorylation (Figure 7). More specifically, the lowest band represented the non-phosphorylated Cx43 isoform (NP-Cx43) while the two other bands represented the phosphorylated isoforms (P-Cx43). All bands were included in the densiometric analysis of Figure 7. As such, 4 GTX chemicals, in particular 2NF, CYCLO, HQO, and HHC, the NGTX chemical CsA and the NC chemical clonidine hydrochloride (CND) downregulated Cx43 protein expression. The NGTX chemical TPA triggered an elevation of Cx43 protein quantities. When assessing the various isoforms separately, NP-Cx43 was the highest expressed in most conditions. However, the expression pattern of the three isoforms could be altered by some compounds. Indeed, the ratio of NP-Cx43 to P-Cx43 was lowered for methapyrilene hydrochloride (MPH), PIPB and TPA while it was significantly enhanced for HHC and TAF (Figure 8). Based on the immunostaining analysis, Cx43 seemed to be lowered by the carcinogenic compounds 2NF, benzo(a)pyrene (BaP), HQO, TAF, MPH, and TPA, but none of these effects were significant (Figure 9 and Figures S10–S13).

The majority of connexin proteins are located at the cell plasma membrane. Up to 3% of the hepatocyte surface can be occupied by gap junctions, resulting in a dotted pattern during immunostaining [41,42]. Nevertheless, connexins can also be detected in the cytoplasm because of their rapid turnover rate [43,44,45]. Cx26 is preferably expressed by periportal hepatocytes, while Cx43 and Cx32 are ubiquitously expressed across the liver acinus [12,46,47]. The staining pattern solely located Cx26 in the cytoplasm of all conditions with 2NF as the only exception (Figure 4 and Figure S3). Here, Cx26 could also be seen as a dotted pattern inside the cell’s nuclei (Figure 4; a detailed view of the protein localization is provided for 2NF to indicate the difference in localization with the other compounds). Cx32 was detected in the intracellular space after exposure to all chemicals (Figure 6 and Figures S5–S8), but additionally displayed a dotted pattern at the cell plasma membrane and/or the nucleus (Figure 6; a detailed view of HQO was provided to indicate the subcellular localization more clearly). Cx43 was mainly detected in a punctuated pattern at the cell plasma membrane (Figure 9 and Figure S10–S13). However, care must be taken when interpreting the immunostaining results, as HepaRG cells combine two different cell types [48]. While membrane-bound Cx32 appeared as punctuation in the cell membranes of the hepatocytes (Figures S5–S8), membrane-bound Cx43 only seemed to be detected in the membrane of the biliary epithelial-like cells (Figures S10–S13). Cytoplasmic Cx43, on the other hand, seemed mostly restricted to the hepatocytes.

### 2.4. Effects of (Non)-Carcinogenic Chemicals on Gap Junction Activity

**Figure 10:**
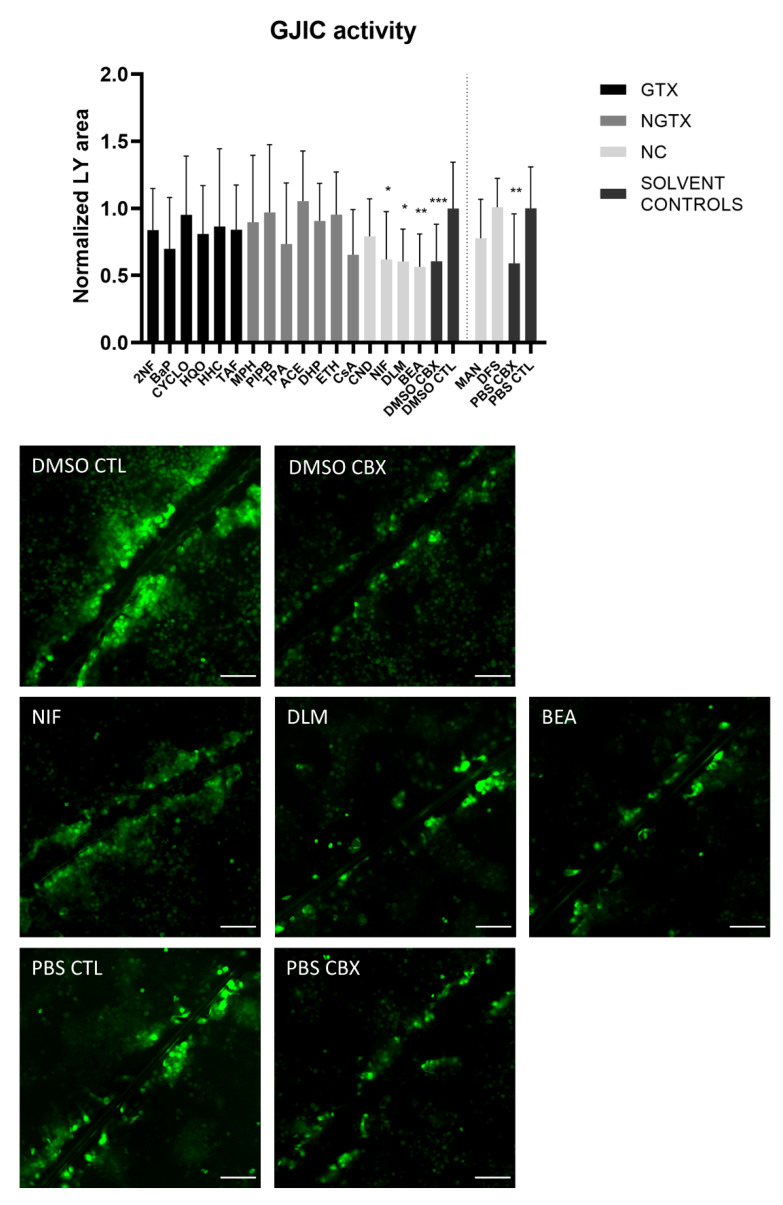
Gap junction intercellular communication in human hepatoma HepaRG cells exposed to GTX, NGTX, and NC chemicals. Human hepatoma HepaRG cells (*n* = 2; N = 2; at least 3 images per well) were exposed to GTX, NGTX, and NC chemicals for 72 h and compared to their respective solvent control. Carbenoxolone disodium salt (CBX), combined with both solvent controls was used as an inhibitor of gap junction intercellular communication (GJIC). The Lucifer Yellow (LY) area in each image was measured and normalized to the mean LY area of the respective solvent control. Significance was calculated with a non-parametric Kruskal-Wallis test followed by a Dunn’s multiple comparison test. Data are expressed as mean ± standard deviation with * *p* ≤ 0.05 ** *p* ≤ 0.01 and *** *p* ≤ 0.001. Scale bar = 100 µM, 20× objective.

A scrape loading/dye transfer assay was used to assess the effects of the chemicals on GJIC. In this assay, a scratch is made in a confluent cell layer in the presence of a Lucifer Yellow (LY) solution. This fluorescent dye is taken up by damaged cells along the scrape and is passed through to the neighboring cells when they display functional GJIC. The resulting fluorescent area is measured for quantification. Carbenoxolone disodium salt (CBX), a general inhibitor of connexin-based cellular communication, is used as a control. Indeed, cells exposed to CBX displayed significantly reduced the gap junction activity compared to their respective medium solvent controls (Figure 10). The scrape loading/dye transfer assay also showed significant lowered GJIC of the HepaRG cells in response to the three NC chemicals NIF, DLM and BEA. None of the GTX or NGTX chemicals affected GJIC.

### 2.5. Effects of (Non)-Carcinogenic Chemicals on Connexin Hemichannel Activity

In contrast to gap junction activity, which has been associated with homeostasis [36], connexin hemichannel activity has been linked to disease progression in various liver pathologies such as fibrosis [49], liver failure [50], and non-alcoholic steatohepatitis [51]. Its role in HCC and carcinogenesis remains to be explored, but the few studies investigating connexin hemichannels in cancer models suggest that they act as tumor promotors [52,53,54]. Therefore, connexin hemichannel-related responses to the (non)-carcinogenic chemicals were investigated via ATP release. Although ATP is transported through a variety of channels, it is widely used as an indicator of connexin hemichannel activity [55,56].

Chemicals may have an inhibitory or an activating effect on connexin hemichannels. To account for both these scenarios, two practical set-ups were used. Both set-ups were very similar except for the buffer, which was used to dissolve the chemicals during the assay, and the presence or absence of the NC CBX solvent control. Thus, chemicals were dissolved in normal calcium buffer (NC buffer) to measure any activating capacity. The NC buffer does not alter the state of the connexin hemichannels and thus keeps the default state of connexin hemichannels, which is closed or flickering [36]. When a chemical activates or opens the connexin hemichannels, this renders an increased ATP signal compared to the NC solvent control. For measuring any inhibiting capacity, chemicals were dissolved in the divalent-free (DF) buffer, which triggers connexin hemichannel opening. An inhibiting effect will result in a low ATP signal even in the presence of the DF buffer. Both set-ups included positive solvent controls (DF DMSO CTL or DF PBS CTL), which represented the condition where all connexin hemichannels were opened and hence the ATP signal was the highest. The second control was the NC control, where connexin hemichannels remained in their closed or flickering state. The third control was the CBX DF control. CBX will inhibit connexin hemichannel activity even in the presence of the DF buffer.

**Figure 11:**
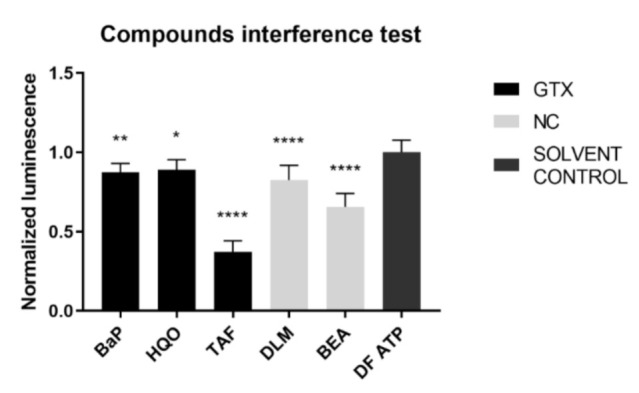
Interference of GTX, NGTX and NC chemicals with the adenosine triphosphate (ATP) luminescence measurement. GTX, NGTC, and NC chemicals were dissolved in a 100 nM ATP solution at their IC_10_ concentration (*n* = 3 and N = 3). Luminescence was measured and data were normalized by the mean luminescence of the DF ATP control. Significant difference compared to the ATP control was calculated with a parametric one-way ANOVA followed by a Dunnett’s post-hoc test to correct for multiple comparisons. Data are expressed as mean ± standard deviation with * *p* ≤ 0.05 ** *p* ≤ 0.01 and **** *p* ≤ 0.0001.

An inherent consequence to the assay set-up is that the compound under investigation was present at the time of the luminescence measurement. To exclude any interference of the chemicals with the read-out, an interference test was performed before execution of the connexin hemichannel assay. BaP, HQO, TAF, DLM, and BEA inhibited the signal and therefore the results of connexin hemichannel assay with these compounds should be interpreted with caution (Figure 11).

**Figure 12:**
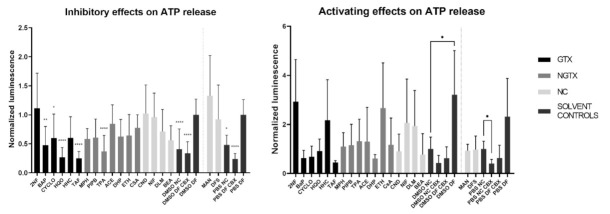
Inhibitory effect or activating effect of GTX, NGTX and NC chemicals on connexin hemichannel-related ATP release in human hepatoma HepaRG cells. Human hepatoma HepaRG cells (*n* = 2–3 and N = 4) were exposed to GTX, NGTX and NC chemicals for 72 h, after which the ATP release assay was performed. DF buffer was used to open the hemichannels, while CBX was used as an inhibitor. Luminescence was measured and data were normalized by the mean luminescence of their respective DF solvent control (assessment of inhibitory effects) or NC solvent control (assessment of activating effects). Significant difference to the respective DF solvent control or NC solvent control was calculated with a non-parametric Kruskal-Wallis test followed by a Dunn’s multiple comparison test. Data are expressed as mean ± standard deviation with * *p* ≤ 0.05 ** *p* ≤ 0.01 and **** *p* ≤ 0.0001.

A total of four GTX chemicals, namely BaP, CYCLO, HQO, and TAF and one NGTX compound, TPA, reduced the extracellular ATP releasecompared to the solvent DF control (DMSO DF or PBS DF) (Figure 12). All compounds except MPH and DHP showed a drop in cell viability compared to the DMSO CTL or PBS CTL, which represented 100% cell viability (Figure S14). This was partly due to the fact that cells were exposed to IC_10_ concentrations of the compounds. However, the assay as such also evoked cell death. Indeed, the DMSO DF condition showed significant lower viability compared to the DMSO CTL condition (Figure S14). When interpreting the results of the hemichannel-mediated ATP release, it is important that cell viability is not significantly different among conditions involved in the analysis. In this respect, 2NF, BaP and HQO caused a significant drop in cell viability compared to the solvent DF control during the assessment of the inhibitory capacity of the compounds (Figure S14, p not shown).

Although ATP release differed a lot among conditions, none of the compounds displayed a connexin hemichannel activating effect compared to their respective NC solvent control (Figure 12). CBX controls were always significantly lowered compared to the DF controls (p not shown). Cell viability was again lowered compared to the DMSO CTL condition for certain compounds, namely 2NF, BaP, CYCLO, ETH, NIF, DLM and BEA (Figure S14). However, when comparing the cell viability to the DMSO NC or PBS NC conditions, only 2NF, HHC and MPH induced a significantly lower viability (Figure S14, p not shown).

## 3. Discussion

Given their key role in maintaining hepatic homeostasis, it is not surprising that gap junctions are frequently involved in liver disease and toxicity [1,57]. In this respect, it has been suggested that gap junctions are specifically negatively affected by NGTX chemicals, but not by GTX chemicals [26,57]. This could have major implications, as currently, unlike for GTX chemicals, no validated in vitro assays are available to identify NGTX chemicals [58]. The present study was set up to verify this hypothesis and simultaneously to investigate whether connexin hemichannels might be more specific targets for NGTX chemicals, thereby using monolayer cultures of human hepatoma HepaRG cells. Although PHH are considered to be the golden standard when it comes to toxicity testing [31]. Human hepatoma HepaRG cells were previously found to be the most appropriate in vitro system among a number of liver-based in vitro models in terms of ability to distinguish GTX chemicals from NGTX and NC chemicals [32]. Despite the fact that HepaRG cells have a tumorigenic origin, they have retained a high differentiation status, which is demonstrated by the presence of functional liver-specific markers such as various cytochrome P450 enzymes and phase 2 enzymes [48]. Additionally, they form colonies of cells displaying a typical polygonal hepatocyte-like shape that are surrounded by biliary-like cells [48]. Overall, HepaRG cells were deemed the best in vitro system to conduct this study, based on these characteristics, the previous in vitro carcinogenicity testing [32] and the fact that PHH display a higher inter-individual variability that can impede reproducibility [59].

Since carcinogenic chemicals can affect liver gap junctions by diverse mechanisms, their effects were investigated at the transcriptional, translational, and activity level of connexin expression after 72 h of exposure. Previous in vitro carcinogenicity testing in HepaRG cells was able to discriminate GTX compounds from NGTX and NC compounds at this time-point [32]. This finding, together with the fact that connexin half-lives are merely a few hours [60,61], indicated that 72 h exposure is ample time to detect alterations in connexin expression and functionality. It was found that Cx32 mRNA expression was lowered by four GTX compounds and one NC chemical (Table 2). This was accompanied by reduced protein expression of Cx32 after exposure to all GTX and NGTX chemicals, which is in accordance with in vivo [4,5,18,62], in vitro [39,63], and ex vivo [37,39,63,64,65,66,67] liver cancer studies. However, this downregulation of Cx32 was not confirmed upon in situ immunostaining. Cx32 is known to relocate to the cytoplasm during HCC [65], yet it was found to concentrate at the cell plasma membrane in HepaRG cells upon exposure to GTX and NGTX chemicals. Cx26, except for 2NF, and Cx43 were not altered by any of the chemicals at transcriptional level. Various effects were triggered by the chemicals at the protein level, but no specific effects related to the type of chemical (i.e., GTX, NGTX, or NC) could be observed. Cx43 has been previously characterized as a tumor promoter in liver [36], yet its expression has been lowered in various HCC models [39,68,69,70] as well as in response to carcinogenic compounds [71,72,73], as is also seen in the present study. This decreased expression has been linked to increased proteasomal activity [71,73].

Based on protein expression and mRNA expression, no clear distinction could be made between NGTX and GTX chemicals in vitro (Table 2). However, gap junction activity is not only dictated by the expression of connexins. Indeed, lowered GJIC has been reported without changes in expression, localization, or post-translational modifications of the connexin building blocks [74]. The scrape loading/dye transfer results did show reduced GJIC for three compounds, yet no link could be made to the type of compounds. Despite the many studies confirming the abrogation of GJIC in response to NGTX chemicals [28], not all NGTX chemicals are reported to inhibit GJIC [26]. Moreover, it has previously been noted that reduced GJIC is not limited to NGTX compounds [75], which is in line with the results of the present study. As such, connexin hemichannels might be more eligible as biomarkers of NGTX activity. Hemichannels are often considered to be pathological pores associated with cell death and inflammation [49,51], which are typical hallmarks of NGTX-mediated carcinogenesis [24]. However, measurement of connexin hemichannel-related ATP release in response to NGTX, GTX, and NC compounds could not substantiate this hypothesis. In fact, various carcinogenic compounds inhibited connexin hemichannel activity, but again no specific effect could be linked to the type of chemicals. Importantly, these connexin hemichannel-related read-outs should be interpreted with caution. To date, it is still technically challenging to specifically monitor connexin hemichannel activity, as they are made out of the same building blocks as gap junctions [53]. Connexin hemichannel assays are therefore still frequently based on non-specific parameters, such as ATP release [55,56].

## 4. Materials and Methods

### 4.1. Reagents and Chemicals

DMSO, LY, CBX, 3-(4,5-dimethylthiazol-2-yl)-2,5-diphenyltetrazolium bromide (MTT), and bovine serum albumin (BSA) were supplied by Sigma-Aldrich (St. Louis, MO, USA). GTX chemicals, NGTX chemicals and NC chemicals (Table 1) were previously selected based on previous in vitro carcinogenicity testing [29,30]. All chemicals were purchased at Sigma-Aldrich (St. Louis, MO, USA) except for ETH, which was obtained at Thermo Fisher Scientific (Waltham, MA, USA). Chemicals to prepare assay buffers were purchased at Sigma-Aldrich (St. Louis, MO, USA).

### 4.2. Cell Viability Assessment

Cell viability was assessed by means of an MTT assay for two purposes. First, inhibitory concentrations of the different chemicals that reduce cell viability by 10%, namely IC_10_ concentrations, were established with an MTT assay during earlier performed in vitro carcinogenicity testing in human hepatoma HepaRG cell cultures [30]. Second, an MTT assay was used in this study to determine cell viability after measurement of connexin hemichannel-related ATP release (see 2.8). For the latter purpose, cells were washed with PBS after exposure to the chemicals and incubated for 90 min at 37 °C with an MTT solution (0.5 mg/mL MTT in Williams’ E medium (Thermo Fisher Scientific, Waltham, MA, USA)). After incubation, the MTT solution was removed and 100 µL DMSO was added to each well to dissolve the formed formazan crystals. Plates were shaken for 10 min and absorption was measured at 595 nm on a VICTOR^3^ Multilabel Plate Counter (PerkinElmer, Waltham, MA, USA). Data were expressed as a ratio to the solvent control cells (DMSO CTL or PBS CTL depending on the respective solvent of the compound; cells exposed to medium containing 0.5% *v*/*v* DMSO or PBS). The use of DMSO or PBS as a solvent was based on preceding in vitro testing [30].

### 4.3. Cell Cultures and Exposure to Chemicals

**Figure 13:**
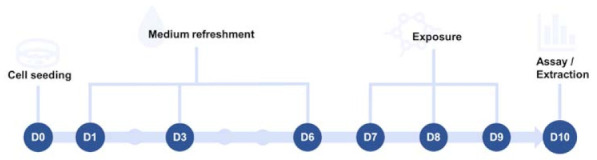
Treatment of cell cultures. The scheme provides an overview of every step in the cell culture process from seeding day (D0) until the final day (D10) when assays or extractions were performed.

PHH were purchased at Kaly-cell (Plobsheim, France). Cryopreserved differentiated human hepatoma-derived HepaRG cells (Biopredic International, Saint-Grégoire, France) were exposed to IC_10_ concentrations of all chemicals for 72 h (Figure 13). Plates were coated with a 0.1 mg/mL collagen solution consisting of rat tail collagen type I (Corning, Glendale, AZ, USA) dissolved in 0.02 N acetic acid (Sigma-Aldrich, St.Louis, MO, USA). Cells were seeded in basal hepatic cell medium (MIL600C, Biopredic International, Saint-Grégoire, France), namely Williams’ E basal medium with GlutaMAX, with HepaRG Thawing/Plating/General Purpose Medium Supplement (ADD670, Biopredic International, Saint-Grégoire, France). Cell culture medium was replaced on days 1, 3, and 6 after seeding with basal hepatic cell medium supplemented with HepaRG Maintenance and Metabolism Medium (ADD620, Biopredic International, Saint-Grégoire, France). Exposure to the chemicals at IC_10_ concentration started at day 7 for 72 h, during which the cell culture medium was replaced daily. Exposure was done in basal hepatic cell medium supplemented with HepaRG Induction Medium Supplement (ADD640, Biopredic International, Saint-Grégoire, France). Chemicals were either dissolved in 0.5% *v*/*v* DMSO or PBS in the final cell culture medium.

### 4.4. Real-Time Reverse Transcription Quantitative Polymerase Chain Reaction Analysis

mRNA expression analysis was carried out as previously described with minor modifications [45]. Total RNA was extracted from cell lysates using an RNeasy Mini Kit (Qiagen, Hilden, Germany) combined with a QIAshredder kit (Qiagen, Hilden, Germany) and an RNase-Free DNase Set (Qiagen, Hilden, Germany) according to the manufacturer’s instructions. The purity and mRNA yield were measured on a NanoDrop 2000 spectrophotometer (Thermo Fisher Scientific, Waltham, MA, USA). Next, cDNA was generated with an iScript™ cDNA Synthesis Kit (Bio-Rad, Hercules, CA, USA) on a MiniAmp Plus Thermal Cycler (Thermo Fisher Scientific, Waltham, MA USA) followed by cDNA purification using the GenElute™ PCR Clean-Up Kit (Sigma-Aldrich, St. Louis, MO, USA). RT-qPCR analysis was performed on a StepOnePlus™ real-time PCR system (Thermo Fisher Scientific, Waltham, MA, USA). Samples were amplified with TaqMan^®^ Gene Expression Assays (Applied Biosystems, Waltham, MA, USA) targeted towards five housekeeping genes, Cx26, Cx32, and Cx43 (Table 3). The reaction mix contained 2 µL cDNA, 1 µL primer, 7 µL nuclease-free water, and 10 µL of TaqMan^®^ Fast Advanced Master Mix (Applied Biosystems, Waltham, MA, USA). Samples were added in duplicate. Amplification efficiency was estimated based on a serial dilution of pooled cDNA from all samples and two no-template controls. Results were analyzed with the 2^-ΔΔCt^ formula in qbase+ (Biogazelle, Ghent, Belgium) and normalized to the DMSO or PBS solvent control.

### 4.5. Immunoblot Analysis

Immunoblot analysis was performed as described previously with small modifications [45]. HepaRG cells were collected by scraping. Following washing steps, the cell pellet was resuspended in radio-immunoprecipitation assay buffer (Thermo Fisher Scientific, Waltham, MA, USA) supplemented with 1% *v*/*v* ethylenediaminetetraacetic acid (Thermo Fisher Scientific, Waltham, MA, USA) and 1% *v*/*v* protease and phosphatase inhibitor cocktail (Thermo Fisher Scientific, Waltham, MA, USA). Next, lysates were sonicated and rotated at 4 °C for 15 min. Supernatants were collected after centrifugation and stored at −80 °C. PHH were not cultured, but freshly thawed for protein extraction. After washing twice with ice cold PBS, cells were pelleted by centrifugating for 5 min at 4 °C at 2060× *g*. The pellet was resuspended in the above-mentioned lysis buffer and vortexed every 5 min while being put on ice for 30 min. The supernatant was collected by centrifugation for 20 min at 4 °C and 14000× *g* and stored at −80 °C. Protein concentrations were quantified using the PierceTM BCA protein assay kit (Thermo Fisher Scientific, Waltham, MA, USA). Equal amounts of proteins (immunoblot analysis of Cx26 and Cx32: 20 µg and immunoblot analysis of Cx43: 10 µg) were separated on 10% or 12% Mini-PROTEAN® TGX Stain-Free™ precast gels (Bio-Rad, Hercules, CA, USA) (Cx26 and Cx32: 12%, Cx43: 10%). For the detection of Cx26, the samples were heated for 5 min at 95 °C before loading on the gel. Following separation, gels were blotted onto a nitrocellulose membrane (Cx43 and Cx32) (Bio-Rad, Hercules, CA, USA) or a polyvinylidene difluoride membrane (Cx26) (Bio-Rad, Hercules, CA, USA). Blocking of the membranes was performed in 5% *w*/*v* non-fatty milk in Tris-buffered saline solution (20 mM Tris and 135 mM sodium chloride) with 0.1% *v*/*v* Tween−20, for 1 h at room temperature. Membranes were incubated overnight at 4 °C with primary antibody diluted in blocking buffer. Primary antibodies were directed against Cx43, Cx32 and Cx26 (Table 4).

Before incubation with the secondary antibody (Cx26 and Cx32: 1:500; Cx43: 1:2000) (P0448 Dako, Glostrup, Denmark) for 1 h at room temperature, membranes were washed three times for 10 min. The secondary antibody was diluted in the blocking buffer. Subsequently, the membranes were washed and visualized using the Pierce™ ECL Western Blotting Substrate kit (Thermo Fisher Scientific, Waltham, MA, USA) on a ChemiDocTM MP imaging system (Bio-Rad, Hercules, CA, USA). Image Lab 6.0.1 software (Bio-Rad, Hercules, CA, USA) was used for densiometric analysis. Signals were first normalized against total protein loading instead of normalization to a housekeeping protein [76] and then expressed as a ratio to the signal of the solvent control.

### 4.6. Immunostaining Analysis

For immunostaining analysis, cells were seeded at a density of 0.48 × 10^6^ cells/well in a 24-well plate (500 µL/well). After washing the cells three times with ice-cold PBS, cells were fixed for 10 min at −20 °C with an ice-cold mixture of acetone and ETH (1:1). Cells were washed again three times after fixation. Next, blocking was performed for 45 min with 1% *w*/*v* BSA/5% *v*/*v* donkey serum at room temperature. Cells were incubated overnight at 4 °C with primary antibody or 1% *w*/*v* BSA dissolved in PBS (control for non-specific binding of secondary antibody). Primary antibodies targeted against Cx43, Cx32, and Cx26 were diluted in 1% *w/v* BSA dissolved in PBS (Table 4). The next day, cells were washed three times with PBS for 5 min, followed by incubation for 1 h at room temperature with the secondary antibody Alexa Fluor 594-AffiniPure Donkey Anti-Rabbit IgG (1:500) (711-585-152 Jackson ImmunoResearch, West Grove, PA, USA) diluted in 1% *w*/*v* BSA in PBS. Cells were washed again with PBS and once with double-distilled water. VECTASHIELD^®^ Antifade Mounting Medium with 4′,6-diamidino-2-phenylindole (DAPI) (Vector Laboratories, Burlingame, CA, USA) was added for staining of the nuclei. Detection was performed on a Nikon Eclipse Ti microscope (20× objective) (Nikon, Tokyo, Japan). Image analysis was performed on Icy software (version 2.1.0.1) [77] and/or ImageJ software (version 1.52p, Bethesda, MD, USA) [78]. Both the fluorescent area and intensity of the fluorescent signal were taken into account and normalized to the number of nuclei. Data were expressed as a ratio to the DMSO or PBS solvent control.

### 4.7. Scrape Loading/Dye Transfer Assay

For the scrape loading/dye transfer assay, cells were seeded in a 24-well plate at a density of 0.48 × 10^6^ cells/well in a 24 well plate (500 µL/well). After 72 h exposure to the chemicals, cells were washed three times with scrape loading/dye transfer buffer (137 mM NaCl, 5.36 mM KCl, 0.8 mM MgCl2, 5.55 mM Glucose and 25 mM 4-(2-hydroxyethyl)-1-piperazineethanesulfonic acid (HEPES); pH 7.4) or CBX buffer (100 µM CBX dissolved in scrape loading/dye transfer buffer). A scratch was made in each well with a fine needle in the presence of LY (1 mg/mL) dissolved in scrape loading/dye transfer or CBX buffer, followed by 10 min incubation at room temperature. Cells were washed four times with Hank’s Balanced Salt Solution (HBSS)/HEPES buffer (0.95 mM CaCl2.2H20, 0.81 mM MgSO4.7H2O, 137 mM NaCl, 5.36 mM KCl, 5.55 mM glucose and 25 mM HEPES; pH 7.4) and fixed for 10 min in 4% *w/v* paraformaldehyde solution. Cells were washed again four times with HBSS-HEPES buffer and visualized on a Nikon Eclipse Ti microscope (Nikon, Tokyo, Japan) with a 20× objective. At least three images were taken along the scrape in each well. The fluorescent area of the dye spread was determined via ImageJ software (version 1.52p, Bethesda, MD, USA) [78]. Data were expressed as ratio to the area of their respective solvent control.

### 4.8. Connexin Hemichannel Assay

Cells were seeded in a 96-well plate at a density of 0.072 × 10^6^ cells/well in 100 µL/well. After 72 h exposure to the chemicals, cells were washed with HBSS-HEPES buffer, called NC buffer. Next, control cells were washed with HBSS-HEPES buffer free from divalent ions (DF buffer) (139.235 mM NaCl, 5.36 mM KCl, 5.55 mM glucose and 25 mM HEPES; pH 7.4) as a control to open all connexin hemichannels or with 100 µM CBX dissolved in NC and/or DF buffer as a control to close all connexin hemichannels. Cells that were exposed to chemicals were washed with DF buffer (assessment of inhibiting properties of chemicals) or NC buffer (assessment of activating properties of chemicals) containing the chemical of interest at IC_10_ concentration dissolved in 0.5% *v*/*v* DMSO or PBS. Next, cells were incubated for 30 min at 37 °C with 100 µL of the respective washing buffer. After 30 min incubation, 50 µL of buffer was transferred to a white opaque well plate containing 50 µL (1 µL ATP assay mix + 49 µL ATP mix dilution buffer) ATP reaction mixture (Adenosine 5′-triphosphate Bioluminescent Assay Kit, Sigma-Aldrich, St. Louis, MO, USA) per well. Luminescence was measured on a VICTOR3 Multilabel Plate Counter (PerkinElmer, Waltham, MA, USA). Data were normalized to the signal of the DF solvent control or the signal of the NC solvent control. After ATP measurements, cell viability was determined by means of an MTT assay.

To exclude interference of the compounds with the ATP read-out, an interference test was performed. In essence, the chemicals were dissolved in a 100 nM stock solution of ATP at their IC_10_ concentration. For each chemical, 50 µL chemical-ATP mixture was transferred to a white opaque well plate containing 50 µL (1 µL ATP assay mix + 49 µL ATP mix dilution buffer) ATP reaction mixture (Adenosine 5′-triphosphate Bioluminescent Assay Kit, Sigma-Aldrich, St. Louis, MO, USA) per well. Luminescence was measured on a VICTOR3 Multilabel Plate Counter (PerkinElmer, Waltham, MA, USA). Data were normalized to the signal of the 100 nM ATP control.

### 4.9. Statistical Analysis

All experiments were performed in three different batches of human hepatoma HepaRG cells (*n* = 3) unless mentioned otherwise. The number of technical replicates (N) is mentioned in the figure legends. Statistical analysis was performed in GraphPad Prism 9 software (GraphPad Software Inc., San Diego, CA, USA) and presented as mean ± standard deviation. Normality was assessed by means of a Shapiro-Wilk normality test. Results were analyzed with a parametric one-way analysis of variance (ANOVA) followed by a Dunnett’s post-hoc test, if data were normally distributed. Data that were not normally distributed were analyzed with the non-parametric Kruskal-Wallis test followed by a Dunn’s multiple comparisons test. Significance levels are indicated according to the following symbols: * *p* ≤ 0.05 ** *p* ≤ 0.01 *** *p* ≤ 0.001 and **** *p* ≤ 0.0001.

## Figures and Tables

**Table 1:** Genotoxic carcinogenic (GTX) chemicals, non-genotoxic carcinogenic (NGTX) chemicals and non-carcinogenic (NC) chemicals. Class, full name, abbreviation, solvent (dimethyl sulfoxide (DMSO), or phosphate-buffered saline (PBS)) and IC_10_ * concentration of the selected compounds are listed.

Class	Full Name	Abbreviation	Solvent	IC_10_ *
GTX	2-nitrofluorene	2NF	DMSO	18 µM
	Benzo(a)pyrene	BaP	DMSO	5 µM
	Cyclophosphamide	CYCLO	DMSO	700 µM
	Hydroquinone	HQO	DMSO	150 µM
	Hydrazine dihydrochloride	HHC	DMSO	860 µM
	2-acetylaminofluorene	TAF	DMSO	40.8 µM
NGTX	Methapyrilene hydrochloride	MPH	DMSO	75 µM
	Piperonyl butoxide	PIPB	DMSO	3.2 µM
	Tetradecanoyl phorbol acetate	TPA	DMSO	35 µM
	Acetamide	ACE	DMSO	5.9 mM
	Diethylhexyl phthalate	DHP	DMSO	10 mM
	Ethanol	ETH	DMSO	10 mM
	Cyclosporine A	CsA	DMSO	2.6 µM
NC	Clonidine hydrochloride	CND	DMSO	0.1 µM
	Nifedipine	NIF	DMSO	40 µM
	*D,L*-menthol	DLM	DMSO	1.2 mM
	Benzyl alcohol	BEA	DMSO	6.5 mM
	Mannitol	MAN	PBS	375 µM
	Diclofenac sodium	DFS	PBS	50 µM

* IC_10_: Concentration of the chemical that reduces cell viability by 10%. Based on previous in vitro carcinogenicity testing in human hepatoma HepaRG cell cultures [29,30].

**Table 2:** Effects of GTX, NGTX, and NC chemicals on connexin expression and functionality in human hepatoma HepaRG cells.

Class	Compound	RT-qPCR Results			Immunoblot Results			Immunostaining Results			Scrape Loading/Dye transfer Assay Results	Connexin Hemichannel Assay	
		Cx26	Cx32	Cx43	Cx26	Cx32	Cx43	Cx26	Cx32	Cx43		INH ^1^	ACT ^1^
GTX	2NF	↓ ^2^	↓	-	↓	↓	↓	-	↓	-	-	-	-
	BaP	-	-	-	↓	↓	-	-	-	-	-	↓	-
	CYCLO	-	↓	-	↓	↓	↓	-	-	-	-	↓	-
	HQO	-	-	-	-	↓	↓	-	↓	-	-	↓	-
	HHC	-	↓	-	-	↓	↓	↑	-	-	-	-	-
	TAF	-	↓	-	-	↓	-	-	-	-	-	↓	-
NGTX	MPH	-	-	-	-	↓	-	-	-	-	-	-	-
	PIPB	-	-	-	-	↓	-	-	-	-	-	-	-
	TPA	-	-	-	↓	↓	↑	-	↓	-	-	↓	-
	ACE	-	-	-	-	↓	-	-	-	-	-	-	-
	DHP	-	-	-	↑	↓	-	-	-	-	-	-	-
	ETH	-	-	-	-	↓	-	-	-	-	-	-	-
	CsA	-	↑	-	-	↓	↓	-	-	-	-	-	-
NC	CND	-	-	-	-	-	↓	-	-	-	-	-	-
	NIF	-	↓	-	-	↓	-	-	↓	-	↓	-	-
	DLM	-	-	-	↓	-	-	-	-	-	↓	-	-
	BEA	-	-	-	-	↓	-	-	-	-	↓	-	-
	MAN	-	-	-	-	-	-	↑	-	-	-	-	-
	DFS	-	-	-	↓	-	-	-	-	-	-	-	-

^1^ INH/ACT: assessment of inhibitory or activating properties of the chemicals on connexin hemichannel-related ATP release, respectively. ^2^ ↓ or ↑: Significant downregulation or upregulation, respectively. Absence of any effect is indicated by ‘-’.

**Table 3:** Primers and probes for RT-qPCR analysis of connexin and housekeeping genes. Assay identification, accession number, assay location, amplicon size, and exon boundaries are listed (GJB1, Cx32; GJB2, Cx26; GJA1, Cx43; ACTB, actin beta; B2M, beta-2-microglobulin; GAPDH, glyceraldehyde-3-phosphate dehydrogenase; HMBS, hydroxymethylbilane synthase; UBC, ubiquitin C).

Gene Symbol	Assay Identification	Accession Number	Assay Location	Amplicon Size (Base Pairs)	Exon Boundary
*GJB1*	Hs00939759-s1	NM_000166.5 NM_001097642.2	1547 1496	63	2
*GJB2*	Hs00269615-s1	NM_004004.5	715	123	2
*GJA1*	Hs00748445-s1	NM_000165.4	1031	142	2
*ACTB*	Hs01060665-g1	NM_001101.3	208	63	2–3
*B2M*	Hs00187842-m1	NM_004048.2	134	64	1–2
*GAPDH*	Hs02786624-g1	NM_001256799.2 NM_001289745.1 NM_001289746.1 NM_002046.5	870 928 822 836	157	7 8 7 8
*HMBS*	Hs00609296-g1	NM_000190.3 NM_001024382.1 NM_001258208.1 NM_001258209.1	1070 972 950 1041	69	13–14 13–14 12–13 12–13
*UBC*	Hs01871556-s1	M26880.1	2173	135	-

**Table 4:** Primary antibodies for immunoblot and immunostaining. Target protein, product code, dilution, and supplier of the selected primary antibodies are listed.

Target	Product Code	Dilution Primary Antibody		Supplier
		Immunoblot	Immunostaining	
Cx26	51-2800	1:250	1:250	Invitrogen, Waltham, MA, USA
Cx32	C3470	1:600	1:500	Sigma-Aldrich, St. Louis, MO, USA
Cx43	C6219	1:4000	1:1000	Sigma-Aldrich, St. Louis, MO, USA

## Data Availability

Data are available upon request.

## Supplementary Materials

All data are available online at https://www.mdpi.com/article/10.3390/ijms222111724/s1.
