# Supplementary material for: Connexin-Based Channel Activity Is Not Specifically Altered by Hepatocarcinogenic Chemicals
Source: Int J Mol Sci. 2021 Oct 29;22(21):11724. doi: 10.3390/ijms222111724 (PMC8584159; doi:10.3390/ijms222111724)
Supplement: Supplementary file 1 [file ijms-22-11724-s001.zip › ijms-1399258-supplementary.pdf]

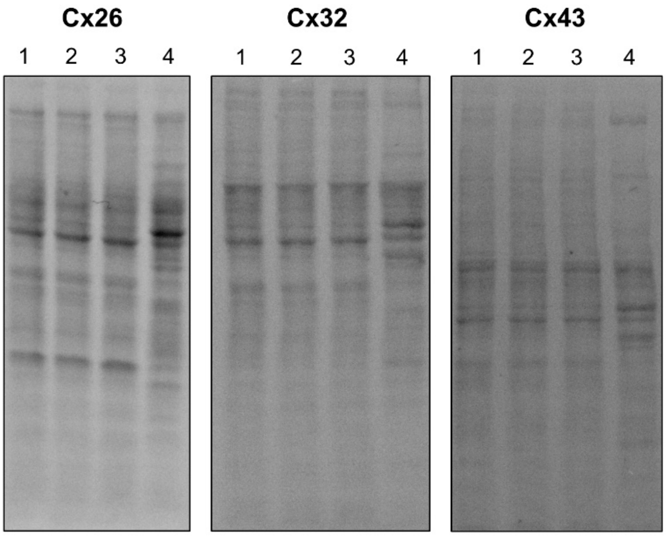


**Figure S1. Total protein loading for normalization of immunoblot analysis.** Before incubation with the primary antibody directed against Cx26, C32 or Cx43, total protein loading was visualized on a ChemiDoc™ MP imaging system (Bio-Rad, Hercules, CA, USA). Image Lab 6.0.1 software (Bio-Rad, Hercules, CA, USA) was used for densiometric analysis. Total protein loading was used to normalize immunoblot signals. (1 = HepaRG batch 284; 2 = HepaRG batch 297; 3 = HepaRG batch 301; 4 = PHH).

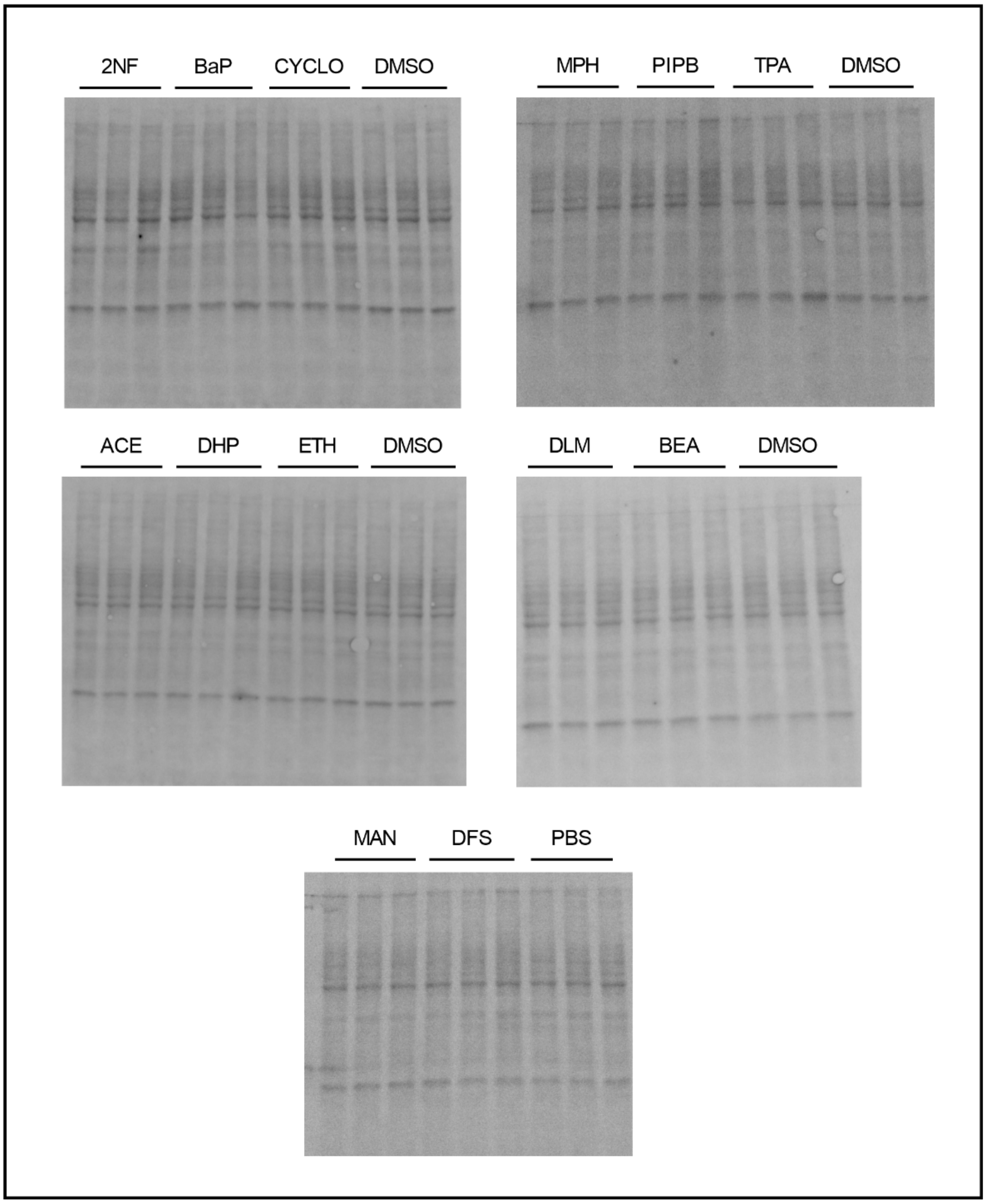


**Figure S2. Cx26 total protein loading for normalization of immunoblot analysis.** Before incubation with the primary antibody directed against Cx26, total protein loading was visualized on a ChemiDoc™ MP imaging system (Bio-Rad, Hercules, CA, USA). Image Lab 6.0.1 software (Bio-Rad, Hercules, CA, USA) was used for densiometric analysis. Total protein loading was used to normalize immunoblot signals.

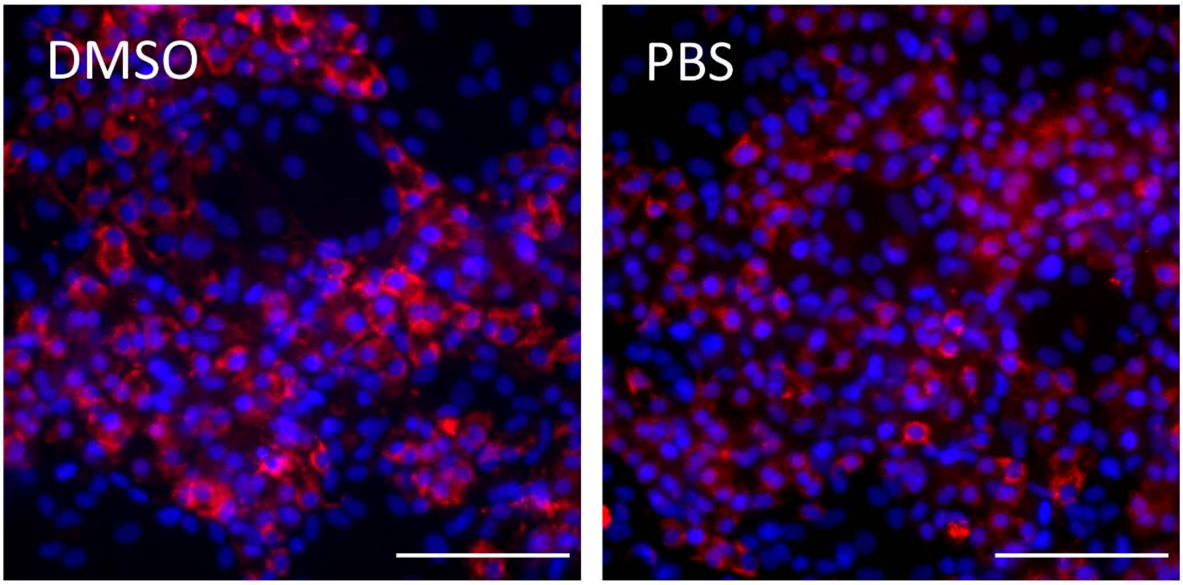


**Figure S3. Detailed Cx26 protein localization in human hepatoma HepaRG cells exposed to DMSO or PBS.** Human hepatoma HepaRG cells (*n* = 1 and N = 1) were exposed to solvent controls (DMSO and PBS) for 72 hours and visualized on a Nikon Eclipse Ti microscope (Nikon, Tokyo, Japan). Scale bar = 100 μM, 40x objective

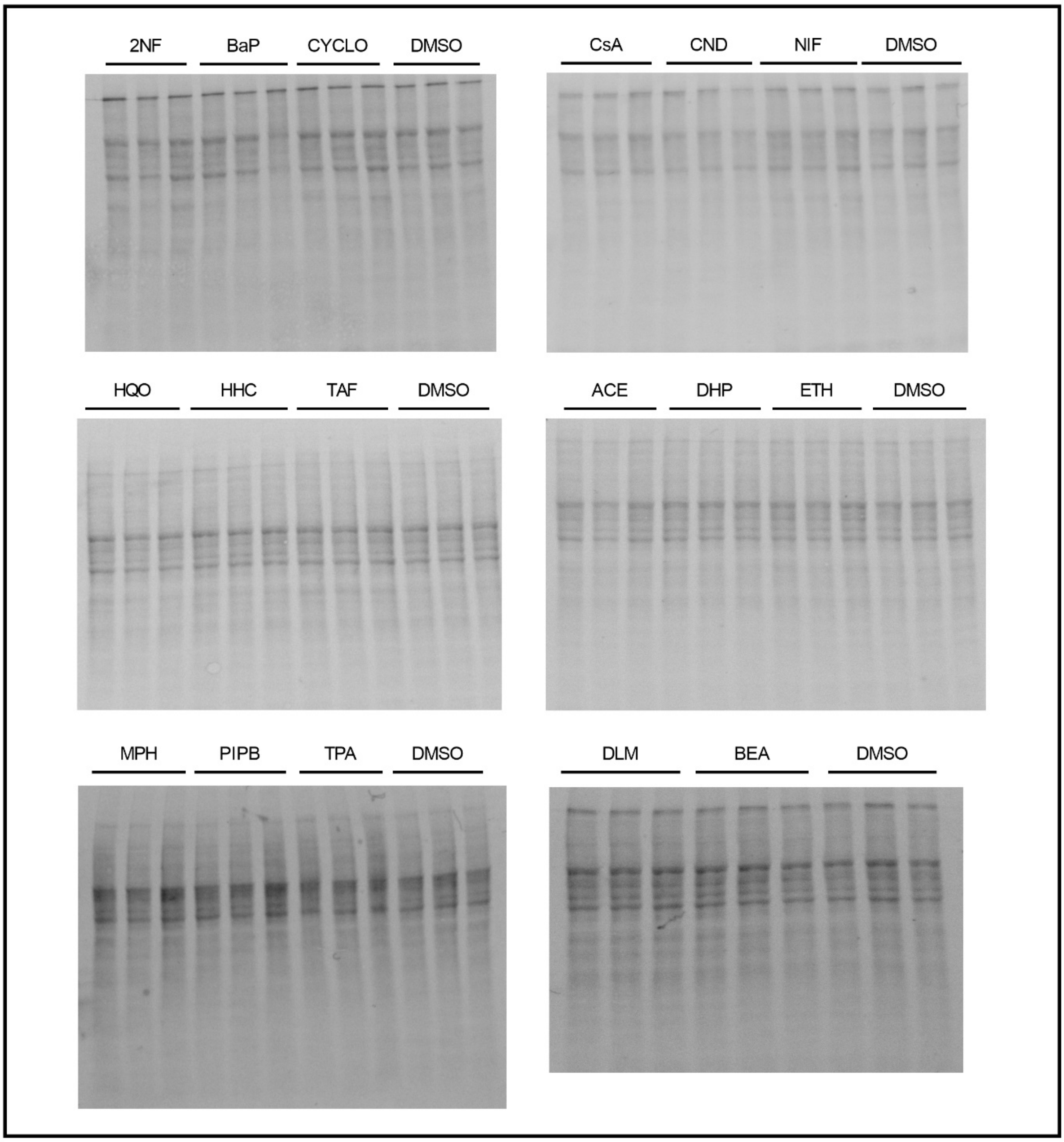


**Figure S4. Cx32 total protein loading for normalization of immunoblot analysis.** Before incubation with the primary antibody directed against Cx32, total protein loading was visualized on a ChemiDoc™ MP imaging system (Bio-Rad, Hercules, CA, USA). Image Lab 6.0.1 software (Bio-Rad, Hercules, CA, USA) was used for densiometric analysis. Total protein loading was used to normalize immunoblot signals

.

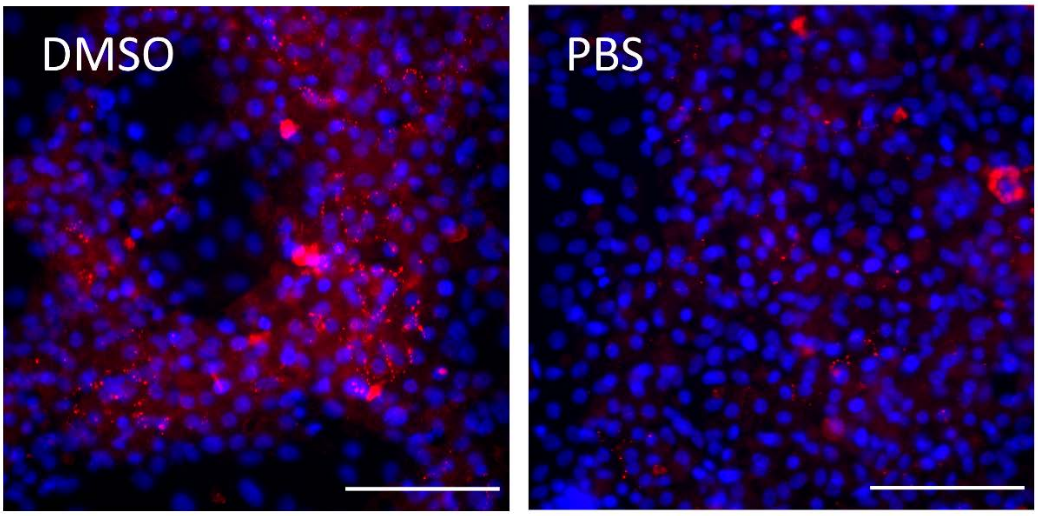


**Figure S5. Detailed Cx32 protein localization in human hepatoma HepaRG cells exposed to DMSO or PBS.** Human hepatoma HepaRG cells (*n* = 1 and N = 1) were exposed to solvent controls (DMSO and PBS) for 72 hours and visualized on a Nikon Eclipse Ti microscope (Nikon, Tokyo, Japan). Scale bar = 100 µM, 40x objective.

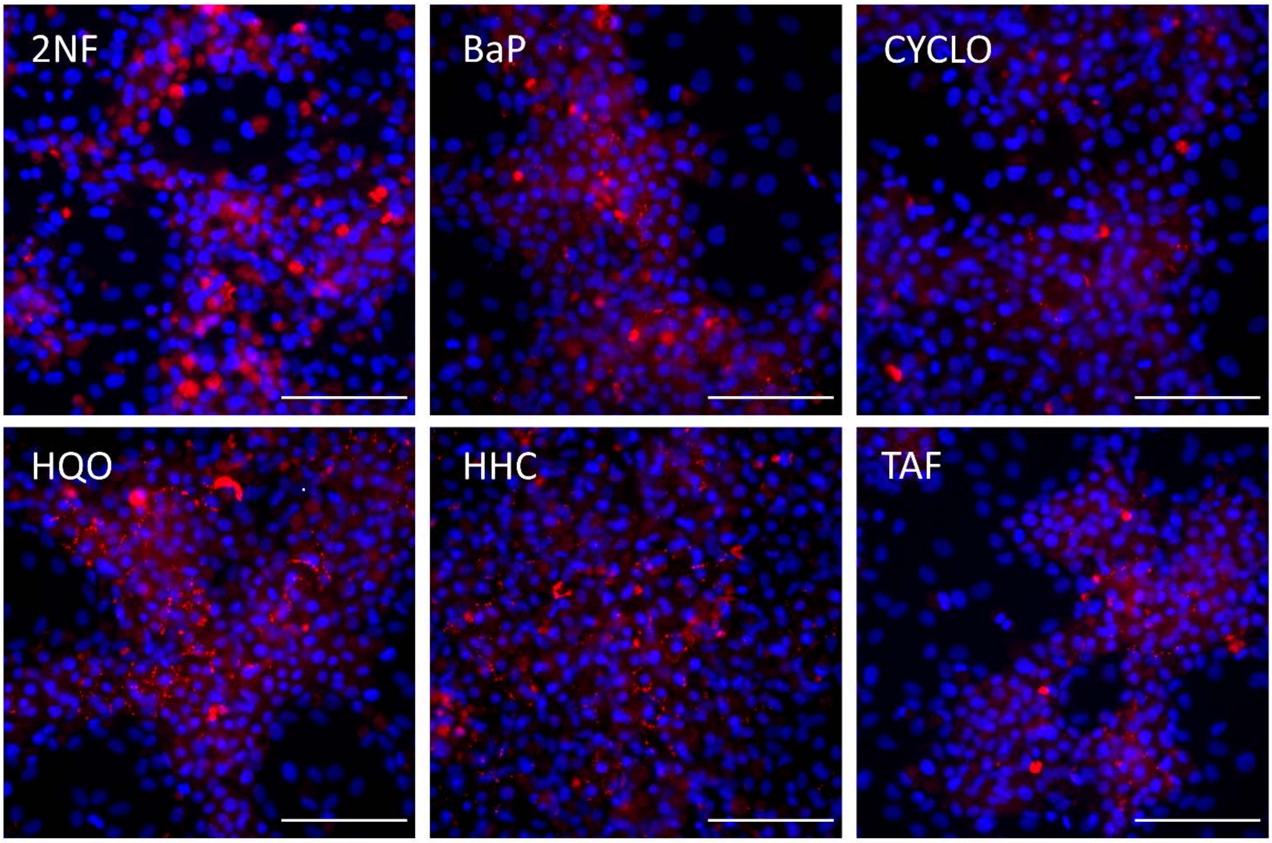


**Figure S6. Detailed Cx32 protein localization in human hepatoma HepaRG cells exposed to GTX chemicals.** Human hepatoma HepaRG cells (*n* = 1 and N = 1) were exposed to GTX chemicals for 72 hours and visualized on a Nikon Eclipse Ti microscope (Nikon, Tokyo, Japan). Scale bar = 100 µM, 40x objective.

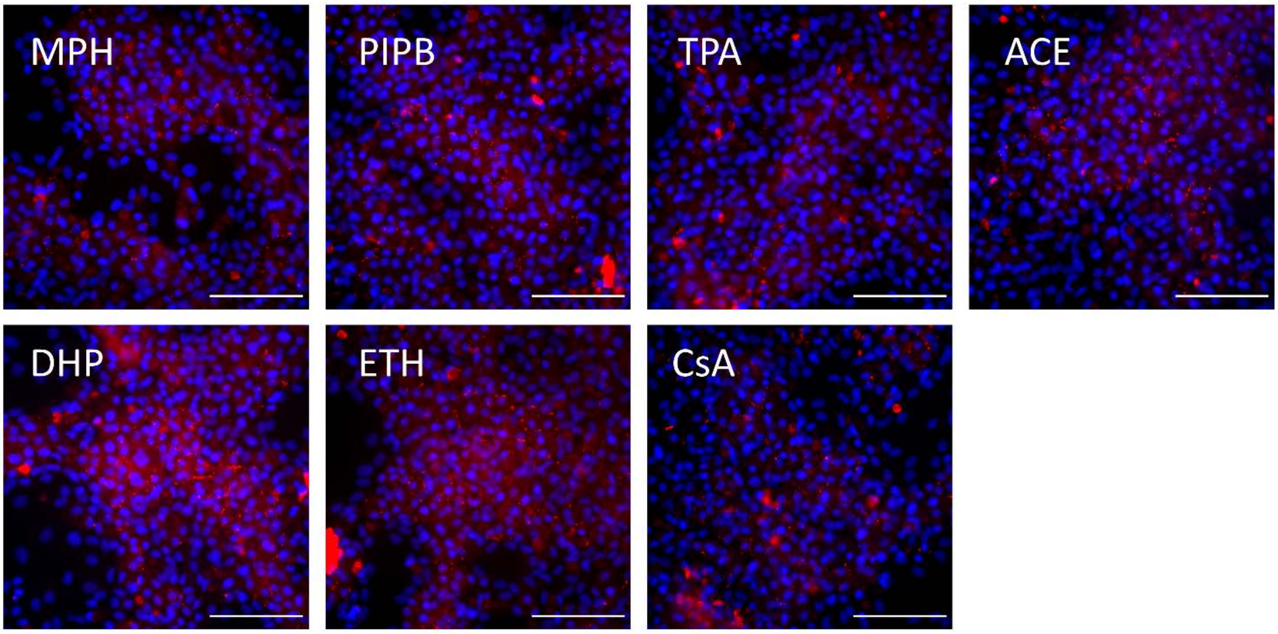


**Figure S7. Detailed Cx32 protein localization in human hepatoma HepaRG cells exposed to NGTX chemicals.** Human hepatoma HepaRG cells (*n* = 1 and N = 1) were exposed to NGTX chemicals for 72 hours and visualized on a Nikon Eclipse Ti microscope (Nikon, Tokyo, Japan). Scale bar = 100 μM, 40x objective.

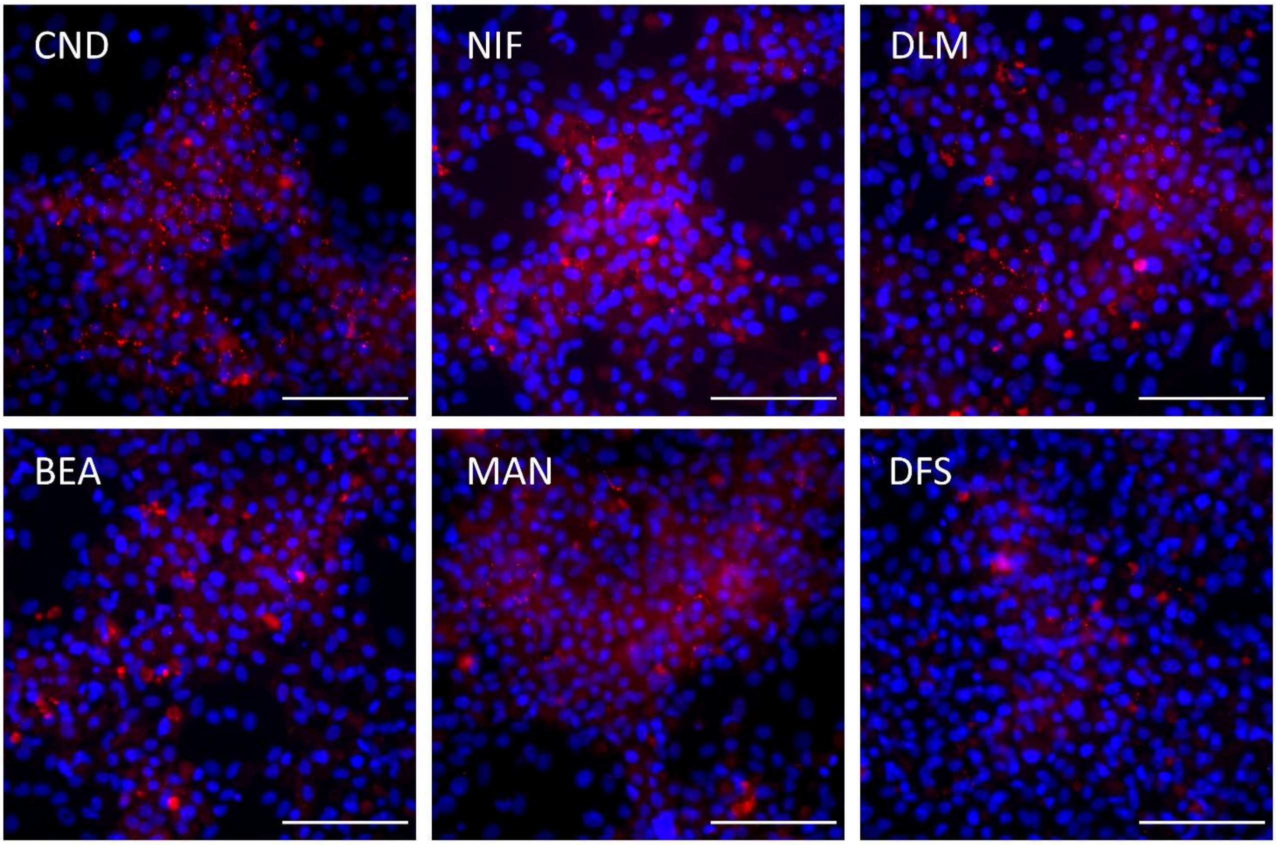


**Figure S8. Detailed Cx32 protein localization in human hepatoma HepaRG cells exposed to NC chemicals.** Human hepatoma HepaRG cells (*n* = 1 and N = 1) were exposed to NC chemicals for 72 hours and visualized on a Nikon Eclipse Ti microscope (Nikon, Tokyo, Japan). Scale bar = 100 μM, 40x objective.

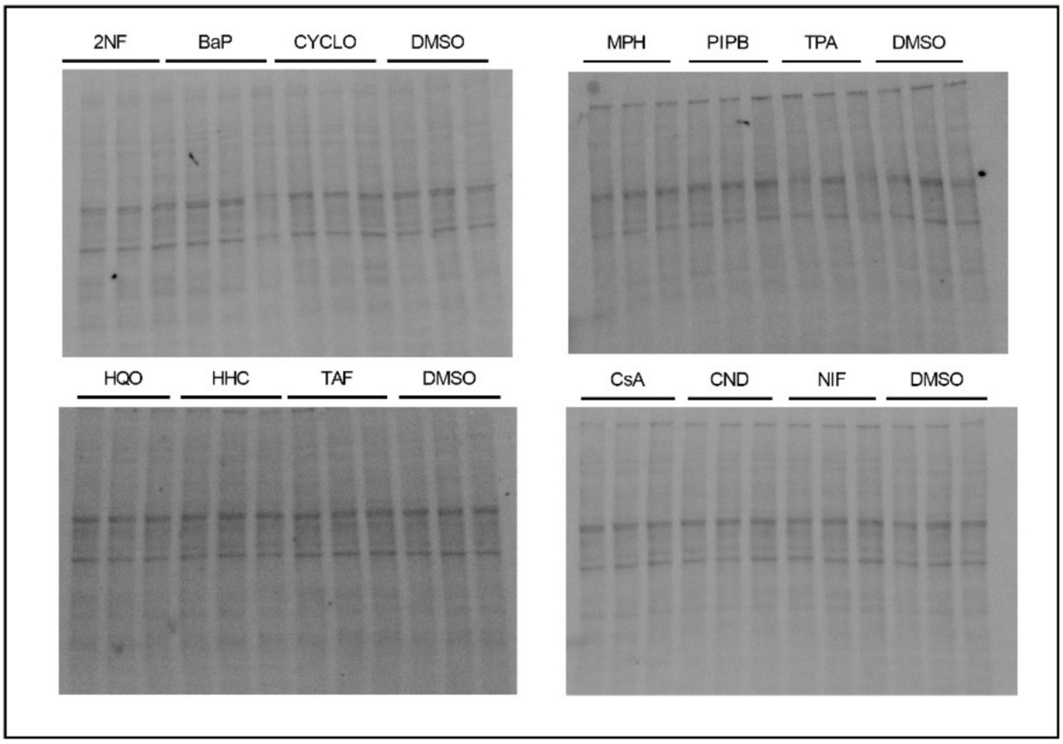


**Figure S9. Cx43 total protein loading for normalization of immunoblot analysis.** Before incubation with the primary antibody directed against Cx43, total protein loading was visualized on a ChemiDoc™ MP imaging system (Bio-Rad, Hercules, CA, USA). Image Lab 6.0.1 software (Bio-Rad, Hercules, CA, USA) was used for densiometric analysis. Total protein loading was used to normalize immunoblot signals.

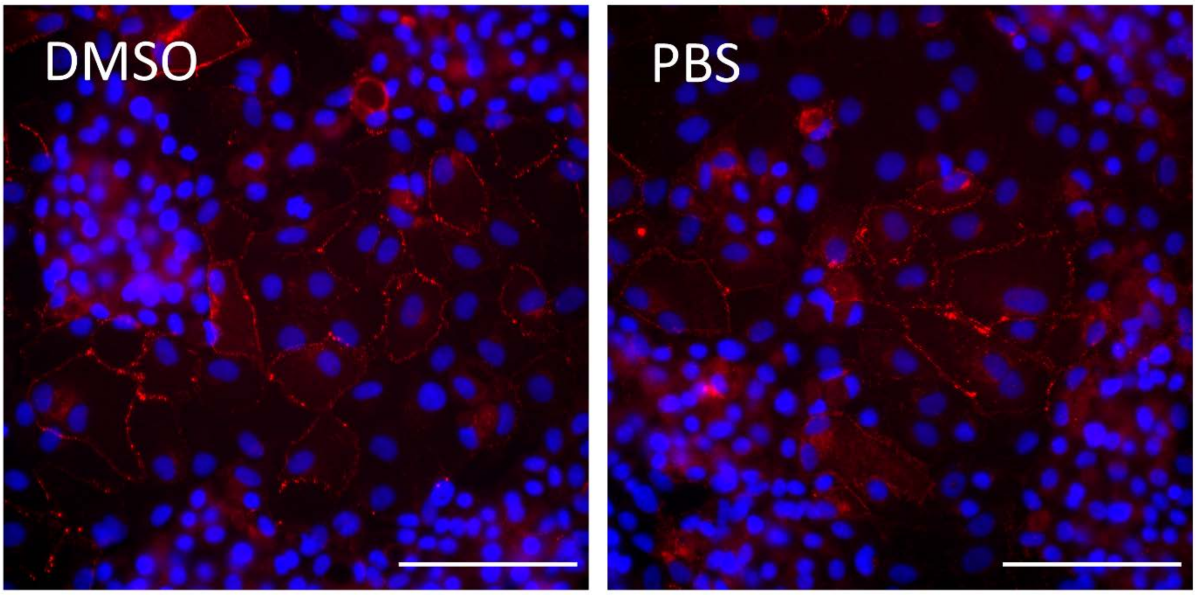


**Figure S10. Detailed Cx43 protein localization in human hepatoma HepaRG cells exposed to DMSO or PBS.** Human hepatoma HepaRG cells (*n* = 1 and N = 1) were exposed to solvent controls (DMSO and PBS) for 72 hours and visualized on a Nikon Eclipse Ti microscope (Nikon, Tokyo, Japan). Scale bar = 100 μM, 40x objective.

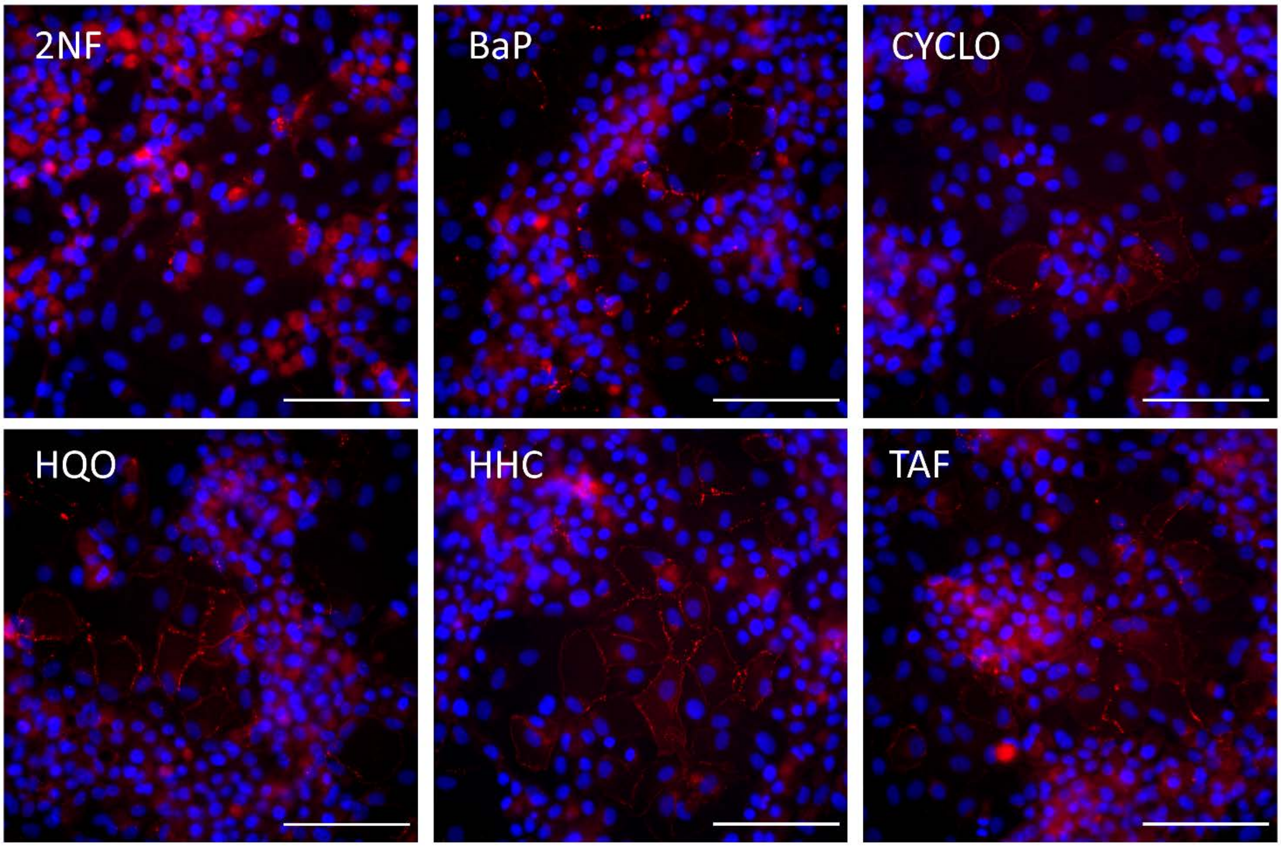


**Figure S11. Detailed Cx43 protein localization in human hepatoma HepaRG cells exposed to GTX chemicals.** Human hepatoma HepaRG cells (*n* = 1 and N = 1) were exposed to GTX chemicals for 72 hours and visualized on a Nikon Eclipse Ti microscope (Nikon, Tokyo, Japan). Scale bar = 100 μM, 40x objective.

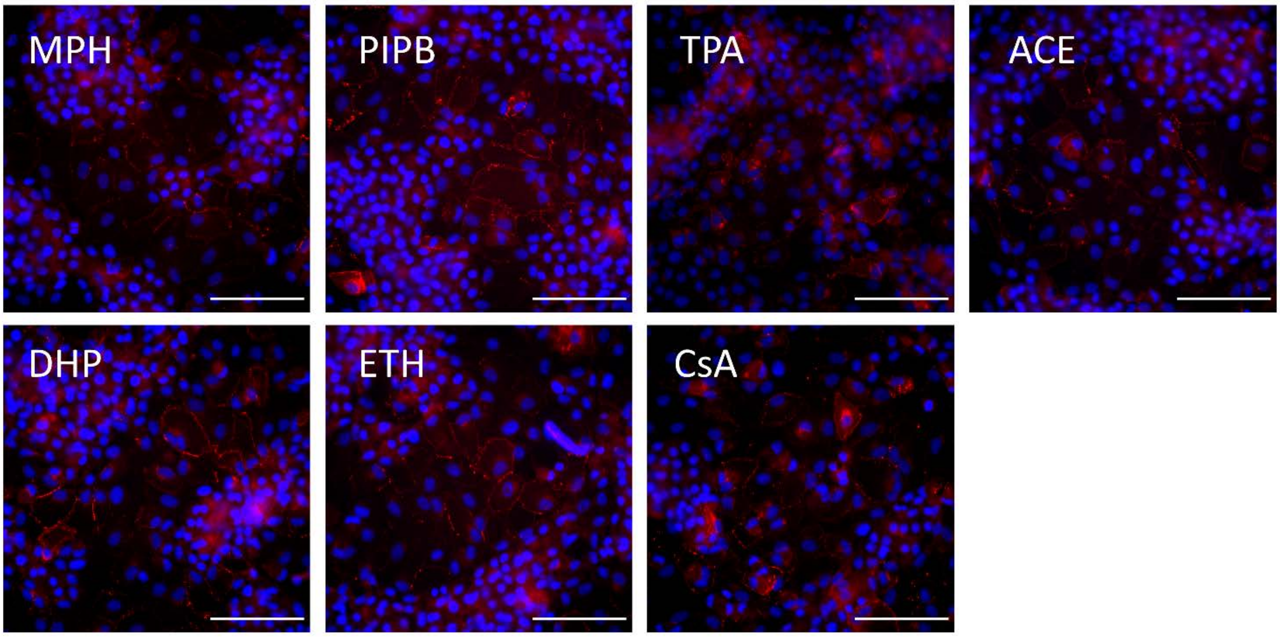


**Figure S12. Detailed Cx43 protein localization in human hepatoma HepaRG cells exposed to NGTX chemicals.** Human hepatoma HepaRG cells ($n$ = 1 and N = 1) were exposed to NGTX chemicals for 72 hours and visualized on a Nikon Eclipse Ti microscope (Nikon, Tokyo, Japan). Scale bar = 100 μM, 40x objective.

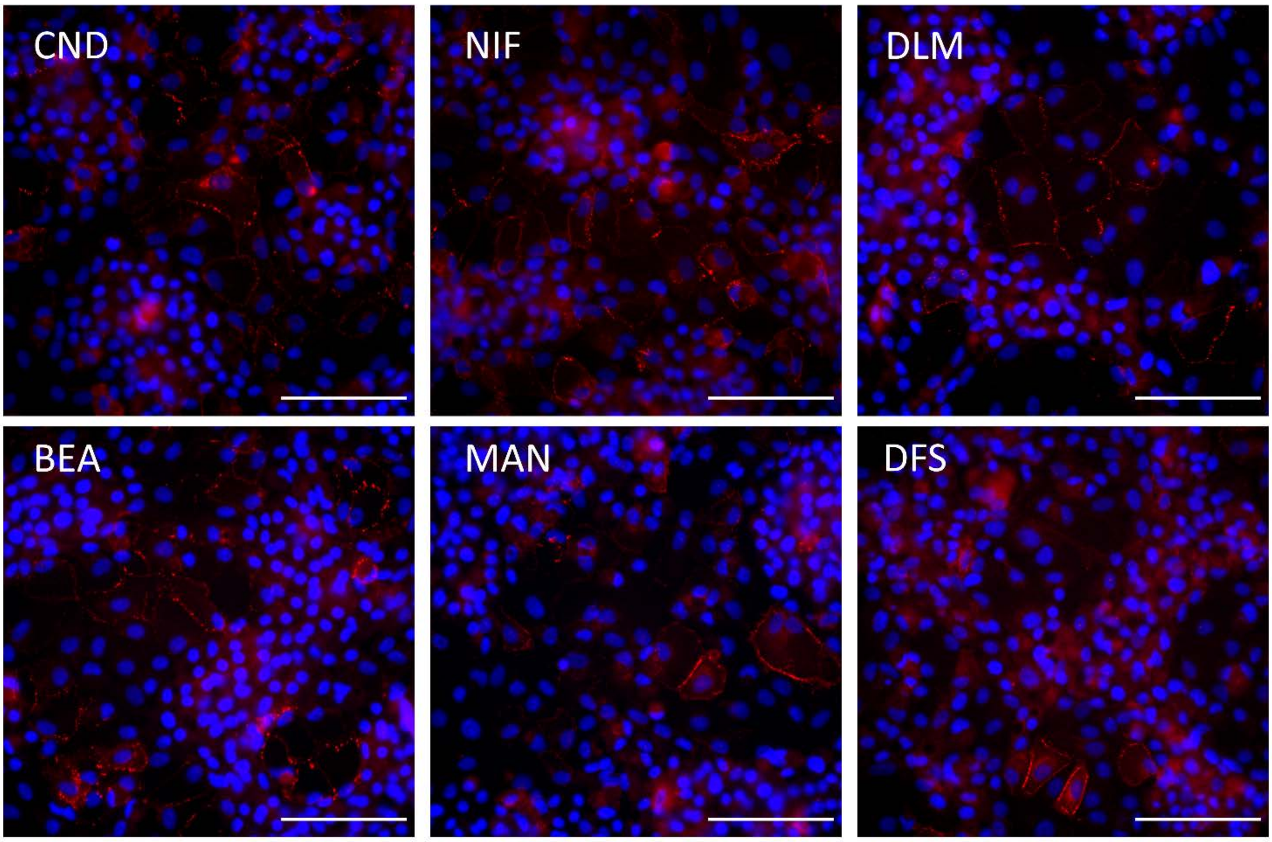


**Figure S13. Detailed Cx43 protein localization in human hepatoma HepaRG cells exposed to NC chemicals.** Human hepatoma HepaRG cells ($n$ = 1 and N = 1) were exposed to NC chemicals for 72 hours and visualized on a Nikon Eclipse Ti microscope (Nikon, Tokyo, Japan). Scale bar = 100 μM, 40x objective.

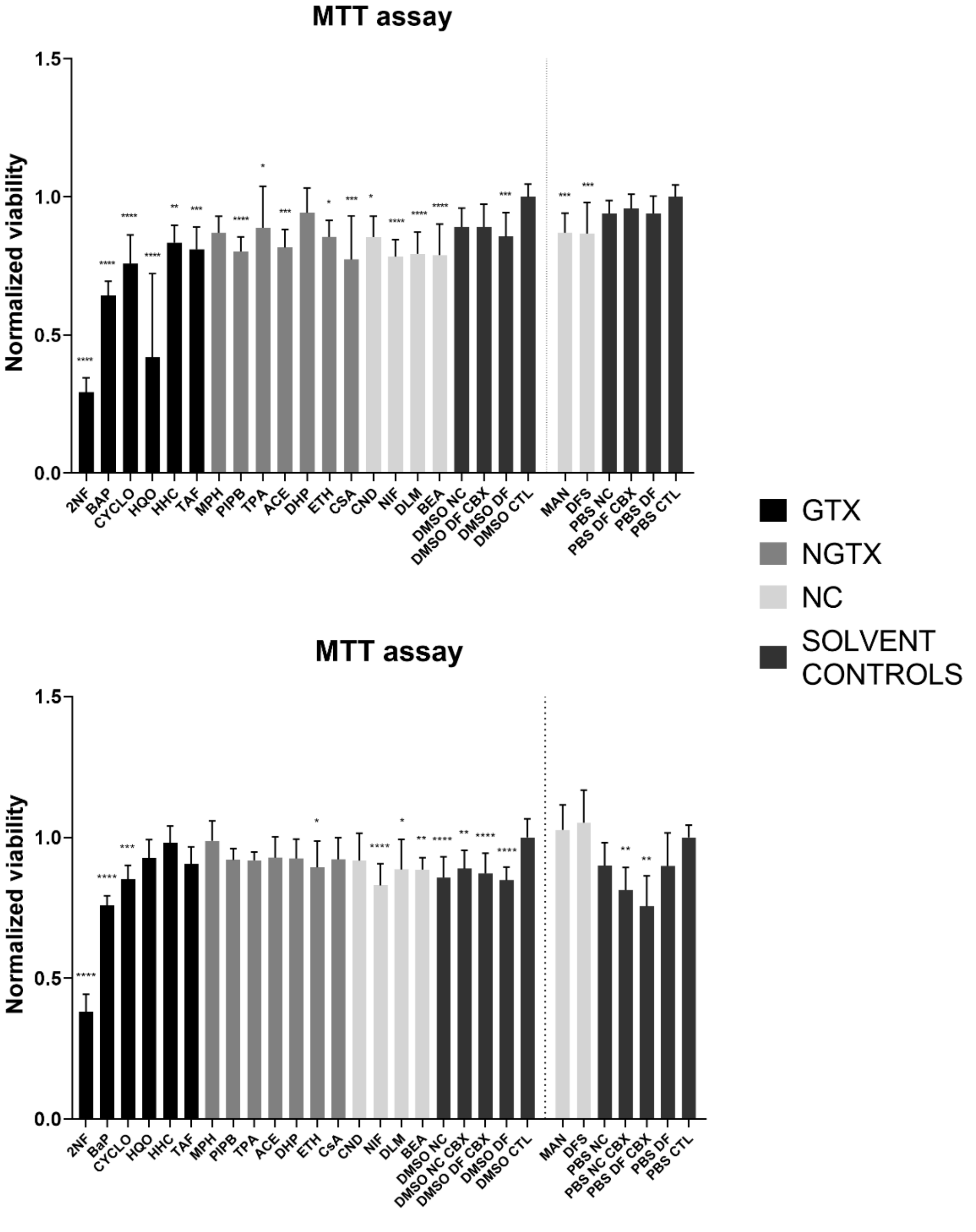


**Figure S14. Cell viability after measurement of connexin hemichannel-related ATP release in human hepatoma HepaRG cells.** Cell viability was assessed after the ATP release assay by means of an MTT assay. Absorbance data were normalized to the mean of the DMSO or PBS CTL, which represents 100% cell viability. Significant difference to the respective DMSO or PBS CTL was calculated with a non-parametric Kruskal-Wallis test followed by a Dunn's multiple comparison test. Data are expressed as mean ± standard deviation with *$p \leq 0.05$ **$p \leq 0.01$*** $p \leq 0.001$ and ****$p \leq 0.0001$.
